# Investigating the Mechanism of Ni-Catalyzed Coupling of Photoredox-Generated Alkyl Radicals and Aryl Bromides: A Computational Study

**DOI:** 10.3390/ijms24119145

**Published:** 2023-05-23

**Authors:** Nil Sanosa, Pedro Ruiz-Campos, Diego Ambrosi, Diego Sampedro, Ignacio Funes-Ardoiz

**Affiliations:** Centro de Investigación en Síntesis Química (CISQ), Department of Chemistry, Universidad de la Rioja, Madre de Dios 53, 26004 Logroño, Spain

**Keywords:** cross-coupling, density functional theory, nickel catalysis, radicals, reaction mechanisms

## Abstract

Photoredox catalysis has emerged as an alternative to classical cross-coupling reactions, promoting new reactivities. Recently, the use of widely abundant alcohols and aryl bromides as coupling reagents was demonstrated to promote efficient coupling through the Ir/Ni dual photoredox catalytic cycle. However, the mechanism underlying this transformation is still unexplored, and here we report a comprehensive computational study of the catalytic cycle. We have shown that nickel catalysts can promote this reactivity very efficiently through DFT calculations. Two different mechanistic scenarios were explored, suggesting that two catalytic cycles operate simultaneously depending on the concentration of the alkyl radical.

## 1. Introduction

Carbon–carbon cross-coupling reactions are one of the most prominent synthetic transformations in organic synthesis, and have a continued and increasing impact in both academia and industry [1,2,3,4]. Since their founding in 1970s [5], the development and extensive application of these reactions are historically attributable to the key role of palladium as a universal catalyst in the various steps of a wide number of strategies such as Heck reactions or Buchwald-Hartwig couplings [6]. However, the high cost and limited availability of palladium are critical challenges that require modifications in the catalytic systems, the most significant of which is the substitution of Pd by other alternative transition metals. In this context, the introduction of non-toxic and earth-abundant metals such as nickel has led to the discovery of new Ni-catalyzed reactions [7], where inactivated organic molecules (alkenes, dienes, alkynes, etc.) become powerful coupling partners [8]. Moreover, the exceptional reactivity of nickel due to its multiple oxidation states (Ni^0^, Ni^I^, Ni^II^, Ni^III^, and Ni^IV^) [9] compared to the most common oxidation states in Pd (Pd^0^/Pd^II^, Pd^II^/Pd^IV^) enables the Single Electron Transfer (SET) processes under suitable reaction conditions, expanding its chemistry to new limits. Thus, the combination of photoredox/metal dual-catalyzed reactions where radicals are generated via SET by the excited photocatalyst paved the way for the extension of the chemical space using milder reaction conditions and even removing the required prefuncionalization steps in the traditional approach [10,11]. Using this methodology, an extensive variety of radical precursors, including alkyl halides [12], carboxylic acids [13], silicates [14], trifluoroborates [15], oxalates [16], and even sp^3^-hybridized C-H substrates via Hydrogen Atom Transfer (HAT) have gradually witnessed its implementation in modern dual photoredox/nickel catalytic systems [17,18].

**Figure 1:**
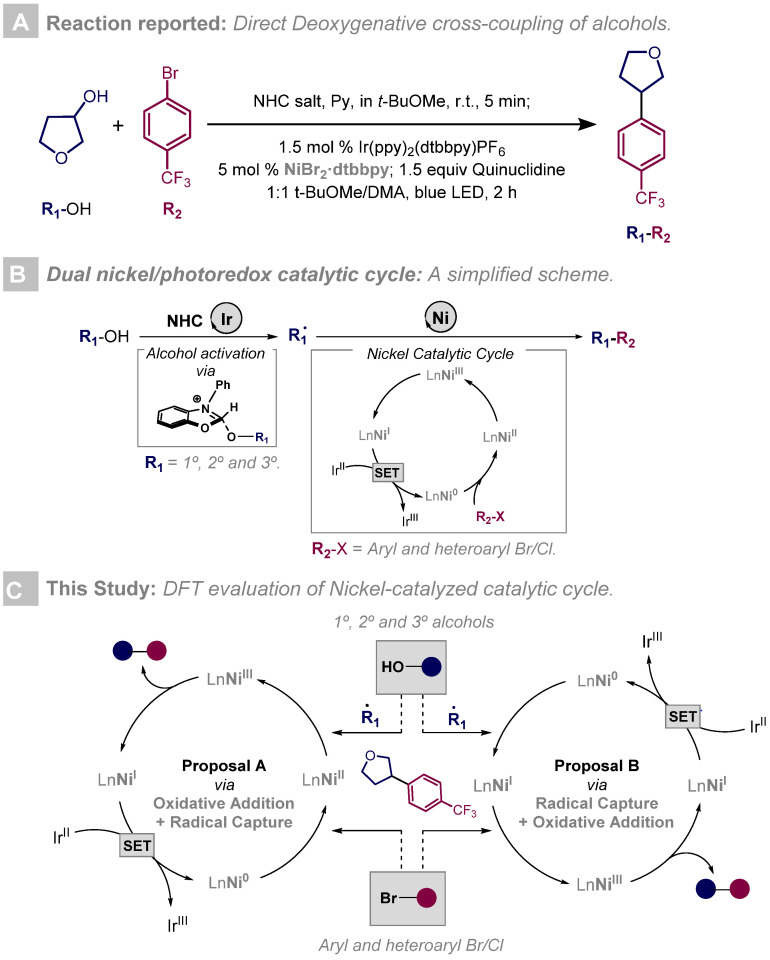
(**A**): Specific reaction conditions reported by MacMillan and co-workers [19]. (**B**): Simplified scheme of metallaphotoredox-enabled deoxygenative arylation of alcohols. (**C**): Proposed nickel catalyzed mechanisms explored in this study.

Within this framework, MacMillan and co-workers recently reported an unprecedented metal catalyzed cross-coupling methodology involving a dual Ni/Ir photoredox catalytic cycle starting from primary, secondary, and tertiary alcohols (Figure 1A) [19]. This strategy grants straightforward access to a wide range of C-C scaffolds using aryl halides as coupling partners. More significantly, this method allows the activation of unreactive C(sp^3^)-OH substrates without requiring previous steps when commercially available N-heterocyclic carbene salts (NHC) are used. This transformation is based on two metal catalytic cycles. To begin with, the activation of alcohol substrates using NHC salts, producing a key alcohol-NHC adduct, leads to the formation of open-shell intermediates via the Ir-photocatalytic cycle (Figure 1B). At this point, the second step of the mechanism involves a nickel-catalyzed system in which Ni^0^ undergoes oxidative addition to an aryl bromide to form an aryl Ni^II^ species. Subsequently, the alkyl radical provided by the Ir-catalytic cycle is trapped, yielding Ni^III^ species. Finally, reductive elimination occurs, delivering the deoxygenative arylation product and completing both the photoredox and nickel catalytic cycles (Figure 1B).

Despite the persistent interest in nickel-catalyzed methodologies over the last few decades, their understanding at the molecular level is still underdeveloped. Unlike the successful computational and experimental characterization of Pd-catalyzed systems [20,21], dual nickel/photoredox catalytic systems contain an inherent complexity that hampers a general understanding of the chemo-, regio-, and stereoselectivity of a great number of protocols. To address this issue, DFT (Density Functional Theory) computational studies have emerged as a reliable tool to shed light on nickel chemistry mechanisms, and we have particularly focused our attention on the flexibility of Ni oxidation states along different reaction mechanisms [22,23]. In this line, the pioneering work by Molander, Kozlowski, and co-workers [24] focused on describing in detail a dual nickel/photoredox catalytic cycle and served as inspiration for further work in this field [25]. Herein, we aim to conduct a comprehensive DFT analysis of the nickel-catalyzed cross coupling of alkyl radicals and aryl bromides reported by MacMillan and co-workers, complementing our initial mechanistic study of alkyl radical generation from alcohols and NHC salts [26]. This DFT study brings up two distinct Ni-catalyzed alternatives, differing on the step where the alkyl radical is trapped by the Ni complex (Ni^II^ vs. Ni^I^) (Figure 1C). This transformation involving a variety of nickel oxidation states will provide valuable insight into organonickel chemistry.

## 2. Results

We initially hypothesized a mechanistic scenario in which the Ni^0^ first engages the aryl bromide in the oxidative addition, leading to a Ni^II^ complex. Subsequently, trapping of the alkyl radical provided by the Ir-photocatalytic cycle produces the key Ni^III^ intermediate, which ultimately delivers the deoxigenative arylation product via reductive elimination (Figure 1C, Proposal A). In addition, we also explored the alternative reaction between the aryl bromide and the Ni^I^ complex, generated upon Ni^0^ trapping of the alkyl radical, to afford the same Ni^III^ complex (Figure 1C, Proposal B). Once the product is formed, the reduction of Ni^I^ to Ni^0^ takes place through SET by the reduced Ir^II^ photocatalyst. In the following sections, a more detailed explanation of the complete Ni-catalytic cycle considering the two alternative mechanisms is described. We first present the initial part of both mechanisms involving Ni^0^ oxidation to Ni^III^ (Section 2.1) and then the reductive elimination step (Section 2.2).

### 2.1. First Part of the Reaction Mechanism: From Ni^0^ to Ni^III^

**Figure 2:**
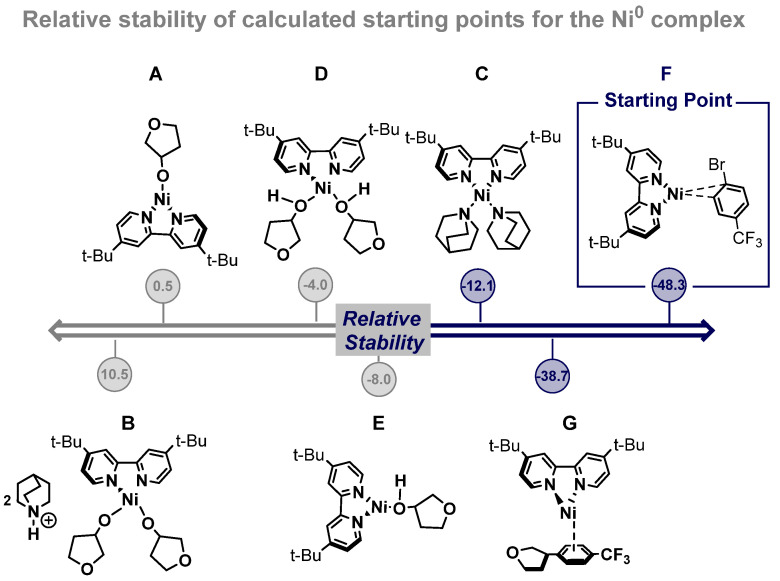
Set of plausible Ni^0^ active complexes (**A**–**G**). Free energies in kcal/mol.

We began our investigation by exploring a suitable Ni^0^ complex as the starting point for both mechanistic proposals. Thus, the Ni^0^ complex could display a coordination number of 3 featuring a trigonal planar geometry or a coordination number of 4 with a square planar geometry. In view of these alternatives, we have studied seven possible starting active Ni^0^ complexes (Figure 2). After analyzing this set of potential Ni^0^ active species, we noticed that compound **F** was clearly the most stable structure, and we set it as our starting point for the nickel catalytic cycle (−48.3 kcal/mol). In addition, product coordination is weaker (−38.7 kcal/mol), so there is no inhibition of the catalyst upon completion of the catalytic cycle.

#### 2.1.1. Mechanism A: Aryl Bromide Oxidative Addition to Ni^0^ and Radical Trapping

**Figure 3:**
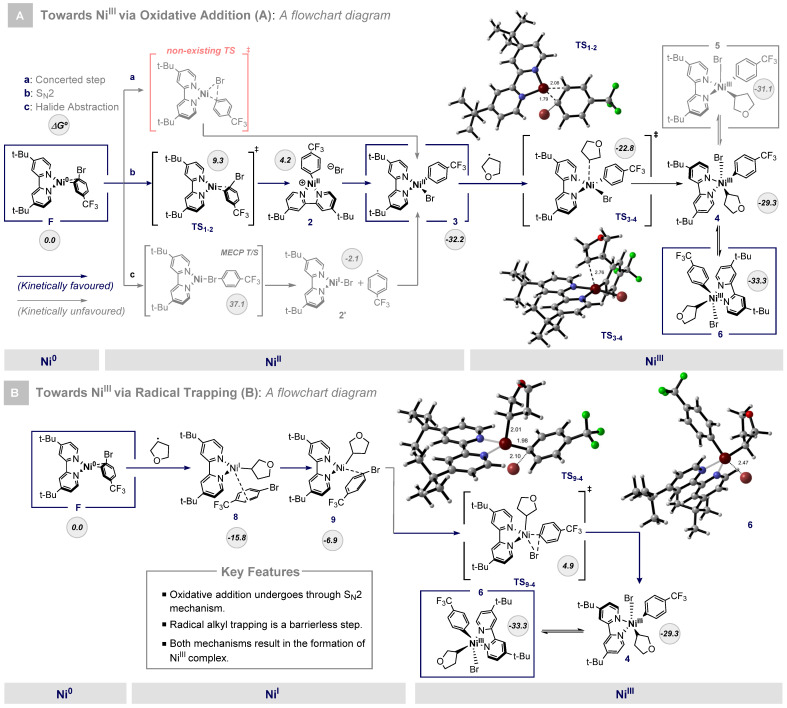
(**A**): Mechanism I is based on a first oxidative addition through the S_N_2 mechanism. (**B**): Alternative mechanism through initial barrierless alkyl radical trapping. Free energies in kcal/mol.

The reaction mechanism begins with the oxidative addition of **F** to yield a Ni^II^ complex. As shown in Figure 3, I, this transformation could occur through three distinct pathways: concerted (a), S_N_2 (b), and via halide abstraction (c). Regarding the concerted mechanism C, the C_sp2_-Br bond is cleaved while the Ni-C as well as the Ni-Br bonds are formed. Otherwise, in the S_N_2 transformation (pathway b), the aryl halide forms a C-Ni bond at the expense of the C_sp_^2^-Br bond cleavage. Finally, the halide abstraction mechanism (pathway c) is based on the Single Electron Transfer of the Ni^0^ to the σ* Br-C_sp_^2^ orbital, resulting in a Ni^I^-Br complex and an aryl radical that can undergo recombination to give **3**. According to DFT calculations, the oxidative addition proceeds easily through a S_N_2 mechanism as it features a readily accessible transition state (**TS_1-2_**, 9.3 kcal/mol). In contrast, a concerted mechanism could not be found as bromide always went away from the Ni center. In addition, the halide abstraction pathway exhibits a very high barrier, so it can be discarded. Thus, surmounting **TS_1-2_** leads to the formation of the cationic intermediate **2**, which is slightly endergonic and can finally trap the bromide ligand and form the square planar Ni^II^ species **3** (−32.2 kcal/mol), a common configuration for d^8^ metals. Once the oxidative addition step was computed, we next evaluated the subsequent transformation consisting of the alkyl radical trapping step. In this way, we envisioned that the alkyl radical could approach the Ni^II^ complex from an axial position, thus minimizing the steric clash between the radical and the Ni^II^ planar geometry (**TS_3-4_**, −22.8 kcal/mol). The structure of this transition state presents a Ni-C_sp3_ bond distance of 2.76 Å and an angle of 104.8º regarding the C_sp3_-Ni-Br atoms. However, upon coordination of the alkyl radical, the Ni-C_sp3_ bond distance decreases to 1.97 Å, and the angle changes to 169.2º, causing the reorientation of the bromine atom to an axial position. We next examined the coordinated isomers of complex **4** to find two different isomers with square pyramidal molecular geometry. In these cases (**5**, −31.1 kcal/mol and **6**, −33.3 kcal/mol), one pyridine ring occupies the axial position due to the weaker coordination with nitrogen.

#### 2.1.2. Mechanism B: Radical Trapping and Aryl Bromide Oxidative Addition to Ni^0^

Alternatively, we explored the radical trapping of Ni^0^ in the resting state. This mechanistic pathway (Figure 3B) begins with barrierless alkyl radical trapping in order to yield the adduct **8** (−15.8 kcal/mol). The subsequent smooth and endergonic reorganization of **8** leads to the formation of the intermediate **9** (−6.9 kcal/mol). At this point, we studied the oxidative addition, and we found the transition state structure **TS_9-4_**_,_ where alkyl is located in an axial position, leaving the equatorial position available for the bromine atom (4.9 kcal/mol), which can take place through a barrier of 21.3 kcal/mol. Finally, after the oxidative addition process is completed through a concerted mechanism, the species **4** (−29.3 kcal/mol), which is shared with the other mechanistic route, is formed. Distinguishing between the two different pathways is not trivial, as alkyl radical concentration is much lower than that of aryl bromide, and then we propose that both pathways can be operating at the same time.

### 2.2. Second Part of the Mechanism: From Ni^III^ to Ni^0^

#### 2.2.1. Reductive Elimination: From Ni^III^ to Ni^I^

**Figure 4:**
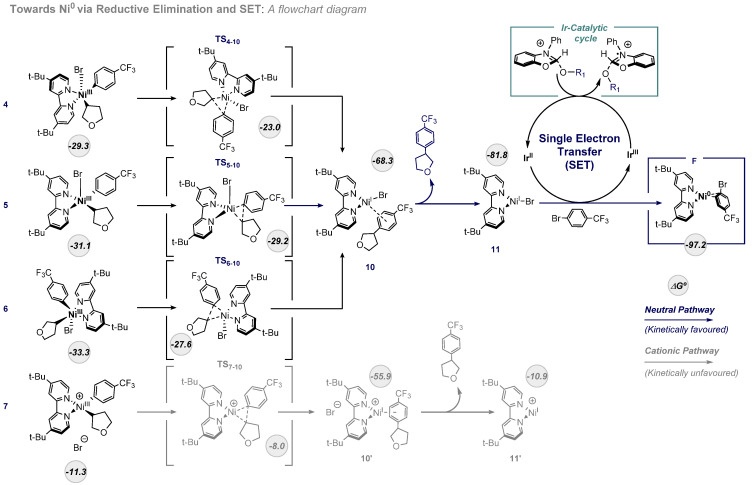
Mechanism of product formation through reductive elimination and SET from Ir^II^ photocatalyst. Free energies in kcal/mol.

The second part of the mechanism (see Figure 4) starts at intermediate **6,** as described above. At this point, the Ni^III^ complex undergoes a reductive elimination to yield the coupling reaction product. We explored all the possible reductive elimination transition states from every Ni(III) intermediate (**TS_7-10_**, **TS_4-10_**, **TS_5-10_**, **TS_6-10_**). The reaction barriers are in all the cases very low (<7 kcal/mol). The last three mentioned transition states will converge into the same intermediate (**10**, −68.3 kcal/mol), while **TS_7-10_** leads to the formation of a cationic intermediate (**10′**, −55.9 kcal/mol). From **10**, the coupling reaction product is released, forming the trigonal planar Ni^I^-Br complex **11** exergonically (−81.8 kcal/mol). This pathway is much more favorable than the formation of intermediate **11′** (−10.9 kcal/mol) through the cationic intermediate **10′**. With the structure of intermediate **11** in hand, we next explored the thermodynamics of the SET process, which reduces Ni^I^ to Ni^0^ and enables the completion of both Ni and Ir catalytic cycles.

#### 2.2.2. Single Electron Transfer (SET): From Ni^I^ to Ni^0^

This last step is based on the SET process catalyzed by the Ir^II^ complex, previously generated as a result of the oxidation of the alcohol-NHC adduct. Thus, intermediate **11** is easily reduced by the presence of the Ir^II^ photocatalyst, which is oxidized to Ir^III^, and the coordination of a second unit of the aryl bromide substrate to regenerate the catalytic cycle. We assume a low SET barrier, as Ir photoredox catalysts are usually very active in SET processes, and the overall thermodynamic free energy change of the reduction of **11** to **F** was calculated considering the oxidation of the photoredox catalyst from Ir(II) to Ir(III). In this way, once SET takes place, the complex species **F** is regenerated (−97.2 kcal/mol), allowing the start of a new Ir/Ni catalytic cycle.

## 3. Discussion

**Figure 5:**
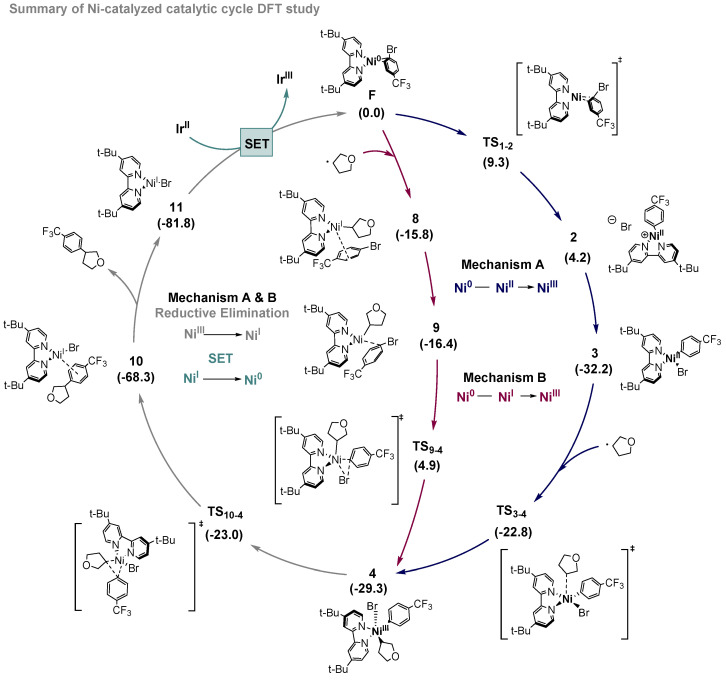
Calculated Ni-catalyzed cycle, including mechanism A (blue) and mechanism B (red). Both mechanisms share reductive elimination and SET processes. Free energy values in kcal/mol.

In summary, we have carried out a DFT computational study of the Ni-catalyzed coupling of alkyl radicals and aryl bromides in the photoredox processes reported by MacMillan and co-workers [19]. This study completes the picture of the whole mechanistic cycle and shows how nickel, as a flexible metal in terms of oxidation states, can engage this reactivity very efficiently, promoting all the steps through low reaction-free energy barriers. We have evaluated two different mechanistic scenarios, and the conclusion is that both can operate simultaneously depending on the concentration of the different species in the reaction mixture. As depicted in Figure 5, both mechanistic proposals converge upon the same Ni^III^ complex, which can deliver the cross-coupling product through an irreversible reductive elimination. The low free energy barriers found are in agreement with the mild reaction conditions used experimentally (room temperature). The rationalization of this Ni catalytic system aims to contribute to the understanding of complex photoredox reaction mechanisms, which involve different catalysts, metals, oxidation states, and radical reactivity, and will then be valuable to create useful models in this still underexplored topic.

## 4. Computational Models

The Gaussian 16 program package [27] was utilized for all DFT calculations. To optimize the structures, the ω-B97xD functional in combination with the DeF2SVP basis set [28] was used. The stationary points were verified as minima (no imaginary frequencies) or transition states (one imaginary frequency) through standard frequency calculations at 298.15 K and 1 atm. Transition states were also verified by relaxing them to reactants and products and performing IRC calculations when needed. Additionally, a single-point calculation was performed using the M06 [29] functional and Def2TZVPP basis set to further refine the potential energies, following the methodology reported before in a related computational study [30]. Parameters for the thermal correction were obtained from the frequency calculations, and the 1M standard state correction was considered by adding 1.89 kcal/mol to all the species. Solvation was included in both optimizations and single-point calculations using the SMD implicit solvent (*N*,*N*-dimethyl acetamide) model [31]. A comparative study including other two different methods is included in the Supplementary Materials. 3D structures were illustrated using the CLYview program [32]. MECP calculations were performed using the easyMECP code [33].

## Data Availability

Cartesian coordinates and energies for all the calculations can be found in the Supplementary Materials.

## Supplementary Materials

The following supporting information can be downloaded at: https://www.mdpi.com/article/10.3390/ijms24119145/s1.
