# Supplementary material for: Investigating the Mechanism of Ni-Catalyzed Coupling of Photoredox-Generated Alkyl Radicals and Aryl Bromides: A Computational Study
Source: Int J Mol Sci. 2023 May 23;24(11):9145. doi: 10.3390/ijms24119145 (PMC10252489; doi:10.3390/ijms24119145)
Supplement: Supplementary file 1 [file ijms-24-09145-s001.zip › ijms-2409630-supplementary.pdf]

Investigating the Mechanism of Ni-Catalyzed Coupling of Photoredox-Generated Alkyl Radicals and Aryl Bromides: A Computational Study

Nil Sanosa, Pedro Ruiz-Campos, Diego Ambrosi, Diego Sampedro *
and Ignacio Funes-Ardoiz *

Centro de Investigación en Síntesis Química (CISQ), Department of Chemistry, Universidad de la Rioja, Madre de Dios 53, 26004 Logroño, Spain
* Correspondence: diego.sampedro@unirioja.es (D.S.); ignacio.funesa@unirioja.es (I.F.-A.)

Table of Contents

Benchmarking Study 3
Intrinsic Reaction Coordinate (IRC) of Two TS 4
Scan Coordinate 5
XYZ Coordinated and Energies of the Calculated Species.. 6

Benchmarking Study

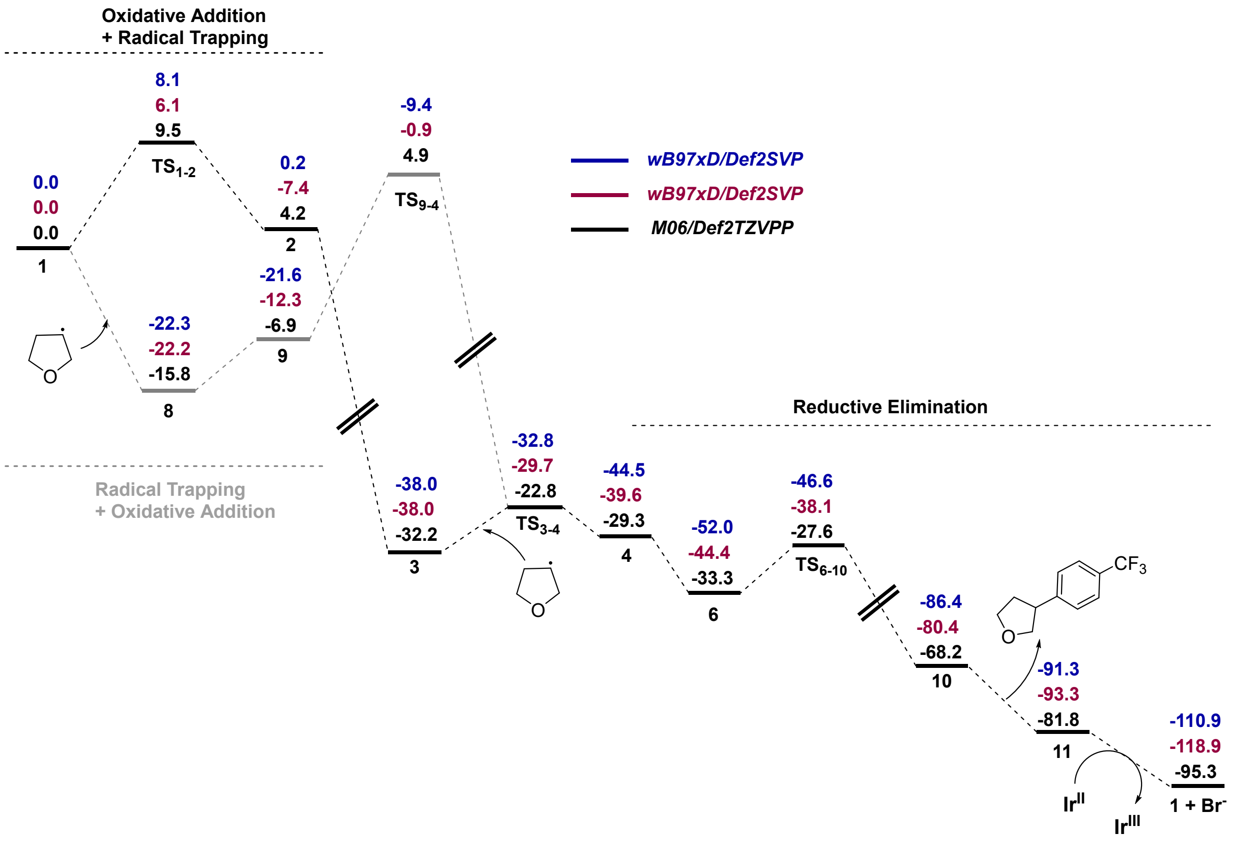

Figure S1: Benchmarking study of SMD (N,N-Dimethyl-Acetamide) wB97xD/Def2SVP (blue), wB97xD/Def2SVPP (purple) and M06/Def2TZVPP (black) on the full free energy profile for nickel catalytic cycle including radical trapping, oxidative addition and reductive elimination. Energy values in kcal/mol.

Intrinsic Reaction Coordinate (IRC) of Two TS

Transition states were further verified by relaxing the imaginary frequency towards the reactant and the product and doing IRC calculations when needed.

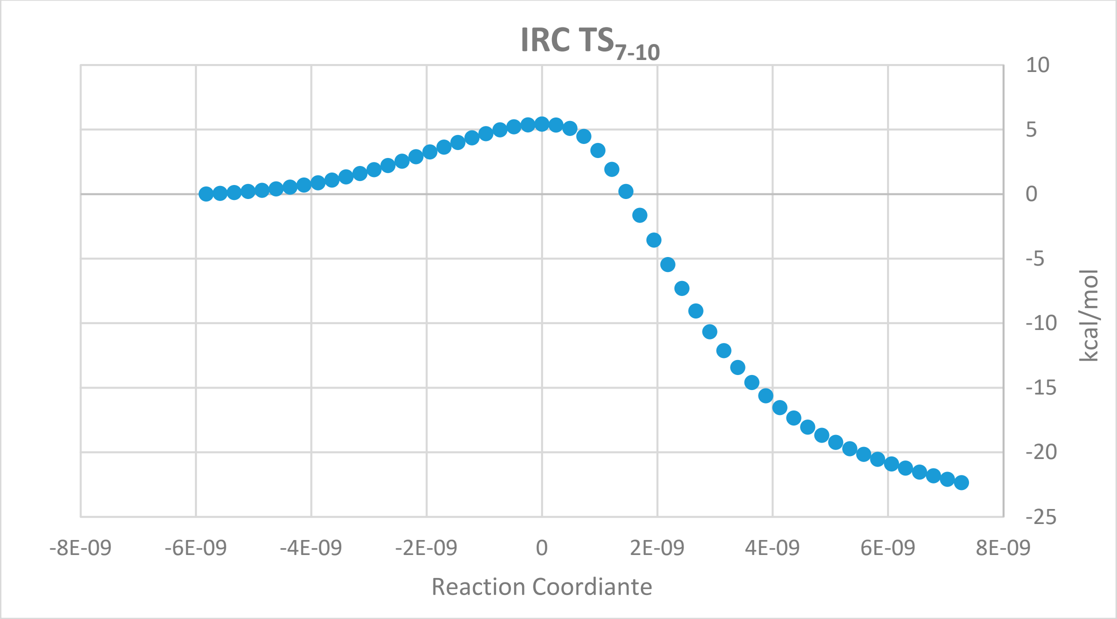

Figure S2: IRC from 7 (reactant) to 10 (product). Energies in kcal/mol.

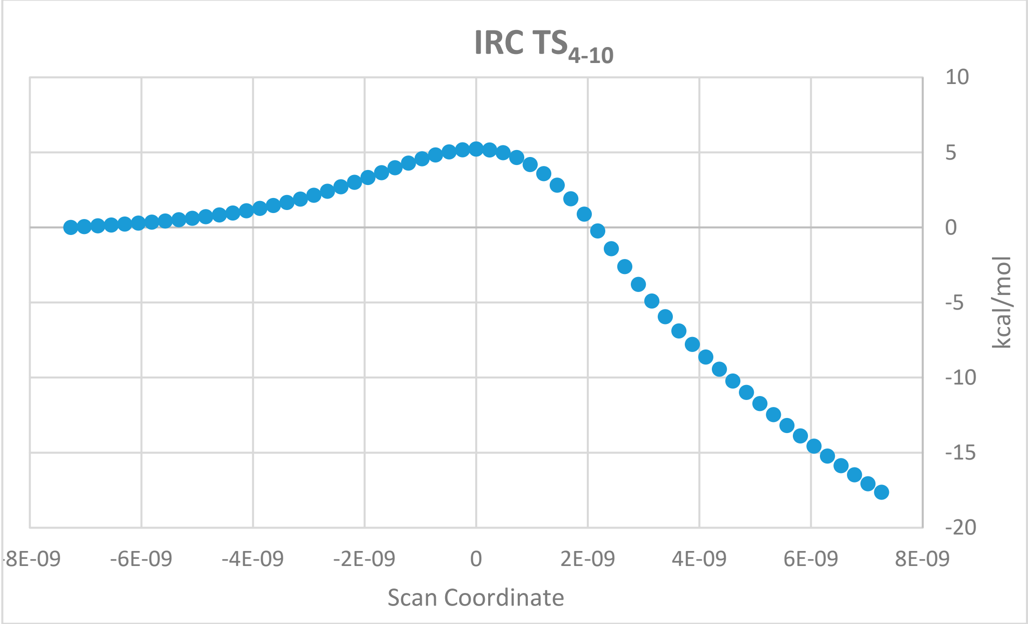

Figure S3: IRC from 4 (reactant) to 10 (product). Energies in kcal/mol.

Scan Coordinate

We ruled out a possible concerted oxidative addition of Ar(C)-Br to Ni^0. We based our point on the following scan coordinate where the energy from **A** (*d(Br-Ni)* = 2.33, *d(Ar(C)-Br)*= 2.95) to compound **B** (*d(Ni-Br)*= 1.85) increases exponentially. For this reason, this pathway does not exist for this specific system.

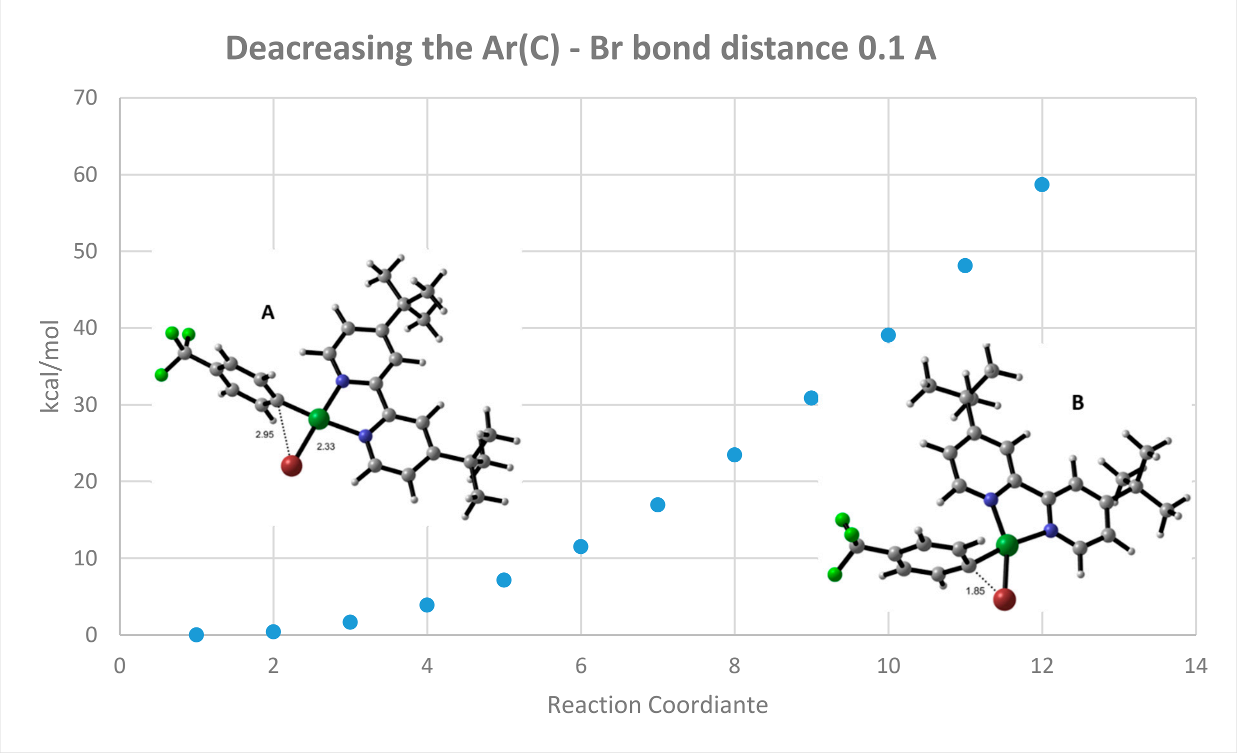

Figure S4: Scan coordinate scheme depicting the Ar(C)-Br bond distance decreasing from A (2.95) to B (1.85). Bond distances are represented in Angstrom and energies in kcal/mol.

Minimum-Energy Crossing Points (MECP)

The resulting energy of MECP appearing to Figure I (34.9 kcal/mol) corresponds to arithmetic average between of the singlet [E(M06)= 5460.55925796] and triplet [E(M06)= 5460.5772203] state providing a final energy number of - -5460.56823913 H. G_{corr} has been calculated using the same equation than before: $G_{corr}(MECP) = [G_{corr}(S) + G_{corr}(T)]/2$, providing a value of 0.409698.

XYZ Coordinated and Energies of the Calculated Species

Final free energies are calculated as the sum of E (basis set employed: M06) + G_{corr}

Q

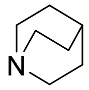

E=-329.183384
G_{Corr}= 0.165436

C	-0.79453300	-0.22545900	1.36242000
C	1.28692800	-0.00005900	-0.00116100
C	0.75831400	-0.23454700	1.42106000
H	-1.20806600	0.56424400	2.00932800
H	-1.20725800	-1.18215100	1.71952500
H	1.13321400	0.55225700	2.09496800
H	1.13386700	-1.19563300	1.80710500
C	-0.79614900	1.29209400	-0.48487900
H	-1.21115400	1.45714400	-1.49169100
H	-1.20860000	2.07944300	0.16558200
C	0.75661300	1.34851500	-0.50841900
H	1.13004300	1.53900200	-1.52725500
H	1.13243900	2.16365700	0.13036400
H	2.38742900	-0.00013700	-0.00219700
C	0.75610200	-1.11407700	-0.91460900
H	1.13047100	-0.96820800	-1.94043500
H	1.13062800	-2.09142300	-0.57066900
C	-0.79663000	-1.06651500	-0.87542800
H	-1.21060100	-0.89717400	-1.88195500
H	-1.21047400	-2.02122400	-0.51434400
N	-1.27797300	0.00007100	0.00111000

QH

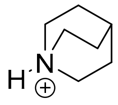

E=-329.656671
G_{Corr}= 0.180864

C	0.74980700	1.40301600	-0.23368500
C	-1.30914700	0.00011100	-0.00285200
C	-0.78508400	1.40170000	-0.33987700
H	1.11786400	1.99874500	0.61071700
H	1.24245700	1.75222000	-1.14898900
H	-1.20614800	2.14250900	0.35365700
H	-1.09625500	1.68341400	-1.35583400
C	0.74619800	-0.49817900	1.33371200
H	1.11557600	-1.52661300	1.42897500
H	1.23468500	0.12077100	2.09550500
C	-0.78891700	-0.40759300	1.38112300
H	-1.20973300	-1.37894100	1.67527400
H	-1.10312000	0.33149500	2.13171500
H	-2.40709500	0.00013000	-0.00526100
C	-0.78350800	-0.99400600	-1.04596200
H	-1.09692000	-2.01407400	-0.78206700
H	-1.20129700	-0.76395700	-2.03573700
C	0.75162400	-0.90510200	-1.09560700
H	1.24163100	-1.87322600	-0.93810800
H	1.12311600	-0.47384800	-2.03309300
N	1.21681500	0.00015400	0.00246500
H	2.24170300	0.00062300	0.00488200

A_1

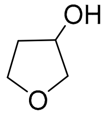

E=-307.606869
G_{Corr}= 0.092535

C	-1.50321400	-0.61193800	0.05547800
O	-1.38206800	0.80295800	-0.06328000
C	-0.02398000	1.17877400	-0.07782200
C	0.77023700	-0.05080900	0.38085800
C	-0.10849500	-1.17902300	-0.16091300
H	-1.88266800	-0.86641200	1.06259800
H	-2.23651700	-0.97823700	-0.68036300
H	0.29937400	1.45825500	-1.10225700
H	0.12847800	2.05439800	0.57353600
H	0.09952300	-1.30869900	-1.23725300
H	0.05263200	-2.14148200	0.34375800
O	2.10969500	-0.06340900	-0.01737100
H	2.13244700	-0.06584800	-0.98285800
H	0.77842100	-0.09038800	1.48244000

A_2

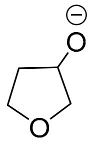

E=-307.081757
G_{Corr}= 0.077693

C	-1.46109200	-0.60558200	0.07913800
O	-1.34042200	0.81282100	-0.01116600
C	0.02423400	1.16580400	-0.12203600
C	0.89452700	-0.05446200	0.31648800
C	-0.08355500	-1.17762900	-0.19497000
H	-1.81335100	-0.88078900	1.09340100
H	-2.23615600	-0.94500200	-0.63198800
H	0.29863000	1.39774900	-1.17509600
H	0.21326500	2.07082900	0.48354900
H	0.10390300	-1.29092600	-1.27850300
H	0.08654800	-2.15290000	0.28860600
O	2.13753900	-0.07284600	-0.07854100
H	0.72554200	-0.08754500	1.46596800

A_3

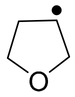

E=-231.720162
G_{Corr}= 0.073443

C	0.83521800	-0.83459100	0.18049600
O	-0.49969400	-1.06824200	-0.22176000
C	-1.24975300	0.09686300	0.06025400
C	-0.26407800	1.21674900	0.04608000
C	1.10290200	0.64582000	-0.11783000
H	0.95785400	-1.03167100	1.26462700
H	1.48957000	-1.52597900	-0.36963900
H	-2.05036200	0.19739400	-0.69620600
H	-1.75940900	0.01519400	1.04778300
H	-0.52192200	2.27467900	0.10935700
H	1.48073100	0.77927600	-1.15148800
H	1.85535800	1.08799700	0.55565500

B_1

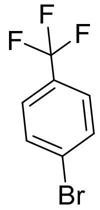

E=-3142.697622
G_{Corr}= 0.059469

C	0.65355200	1.22446700	-0.00167200
C	-0.74014200	1.22906900	-0.00286300
C	-1.44077000	0.02469100	-0.00351400
C	-0.75267700	-1.19157500	-0.00282200
C	0.63759400	-1.20580200	-0.00177600
C	1.32921700	0.00650400	-0.00105900
H	1.20059200	2.16851500	-0.00114200
H	-1.27284700	2.18098100	-0.00361700
H	-1.29928300	-2.13730100	-0.00385400
H	1.17389500	-2.15599700	-0.00214300
Br	3.22313300	-0.00660600	0.00095600
C	-2.94598000	0.00368400	0.00023900
F	-3.42985100	-0.59381100	1.09902100
F	-3.43378400	-0.67615100	-1.04727200
F	-3.47600400	1.22871700	-0.04529300

B_2

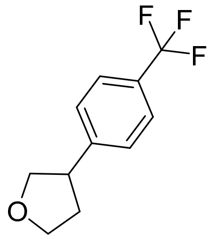

E=-800.420919
G_{Corr}= 0.162792

C	-4.62671800	-0.21396200	0.63398100
O	-4.51889800	-0.25353800	-0.78179900
C	-3.17942300	-0.05082800	-1.17997800
C	-2.44901700	0.50769400	0.04895500
C	-3.20096300	-0.21971700	1.17119700
H	-5.16155200	0.70485600	0.93862300
H	-5.21630600	-1.07716100	0.98258500
H	-2.72347500	-1.00715300	-1.50206800
H	-3.14863900	0.64170900	-2.03653300
H	-2.82997000	-1.25186500	1.27406500
H	-3.10762500	0.27883600	2.14605700
H	-2.67474600	1.58343100	0.12294200
C	-0.95065300	0.33834500	0.02792500
C	-0.11045000	1.45721100	0.03891300
C	-0.36198700	-0.93416600	-0.01416600
C	1.27554300	1.31858100	0.01323400
H	-0.54796800	2.45828700	0.06794500
C	1.01979100	-1.08417700	-0.04149200
H	-0.99129100	-1.82748900	-0.02742000
C	1.84255000	0.04528800	-0.02755100
H	1.91110600	2.20605500	0.02207700
H	1.45726900	-2.08403900	-0.07564100
C	3.33628600	-0.12117600	-0.00300500
F	3.97609800	0.94461300	-0.49915300
F	3.79876600	-0.29836700	1.24620900
F	3.73787200	-1.18576800	-0.70997900

B_3

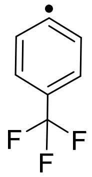

E=-568.542661
G_{Corr}= 0.058099

C	0.11182600	0.02061800	-0.00891200
C	0.78393200	-1.20782900	-0.00557300
C	2.18144300	-1.23679300	0.00097700
C	2.82094100	-0.01572500	0.00384200
C	2.21429500	1.21902000	0.00096900
C	0.81320400	1.22801800	-0.00539200
H	0.22185600	-2.14515600	-0.00842000
H	2.72070900	-2.18730900	0.00294200
H	2.77708300	2.15584200	0.00309400
H	0.27741100	2.17894200	-0.00820000
C	-1.39341800	0.00991500	-0.00092000
F	-1.91732200	1.23442600	-0.10649500
F	-1.87971100	-0.52732600	1.12846200
F	-1.89078900	-0.71884000	-1.01078500

B_4

$Br^{\ominus}$

E=-2574.223587
G_{Corr}= -0.016176

Br	0.00000000	0.00000000	0.00000000

Ir^{II}

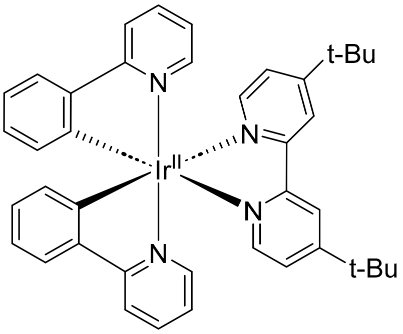

E=-1871.111043
G_{Corr}= 0.633024

C	-1.22905400	-2.01160300	1.15537800
C	-1.93221900	0.05106600	0.39654400
C	-2.52904300	-2.42276600	1.39043500
H	-0.38625200	-2.67143100	1.36845400
C	-3.26654700	-0.30904900	0.59450500
H	-2.69349600	-3.42196000	1.79649400
H	-4.04875600	0.40622600	0.35198400
C	0.23010400	2.76009300	-0.71026000
C	-1.52251700	1.39532100	-0.08143200
C	-0.61561700	3.83729100	-0.90717900
H	1.30450700	2.85189700	-0.88238900
C	-2.42848900	2.44357800	-0.26092500
H	-0.18432700	4.78371100	-1.23703400
H	-3.48224300	2.27093400	-0.05524700
N	-0.92610000	-0.81449600	0.63565900
N	-0.20523900	1.55570900	-0.31430400
C	-1.99414000	3.69748700	-0.68978200
C	-3.59976000	-1.57463800	1.07779100
C	-5.04281100	-2.05058700	1.24851600
C	-6.05559100	-0.96089500	0.88149400
C	-5.26116800	-3.26249900	0.32382000
C	-5.27043000	-2.46636500	2.71204200
H	-5.94838800	-0.06925200	1.51864600
H	-5.96044200	-0.64819600	-0.16984400
H	-7.07671500	-1.34719000	1.02015100
H	-4.59081900	-4.09736100	0.57892400
H	-6.29753400	-3.62445800	0.41423600
H	-5.08427000	-2.99230600	-0.72934600
H	-6.30816000	-2.80928200	2.84787800
H	-4.60603200	-3.28967500	3.01460300
H	-5.09939900	-1.61939400	3.39512700
C	-2.94263900	4.87471900	-0.92410400
C	-2.83814800	5.30412400	-2.39864700
C	-4.39946900	4.50909400	-0.62045600
C	-2.52502200	6.04252300	-0.01316100
H	-1.81986200	5.62857000	-2.66119300
H	-3.11773800	4.47803000	-3.07139900
H	-3.51881200	6.14777200	-2.59372900
H	-4.53826600	4.21051400	0.43024700
H	-5.04272100	5.38288400	-0.80440500
H	-4.76050200	3.69115500	-1.26305200
H	-3.20129900	6.89828600	-0.16589100
H	-2.57551200	5.75345600	1.04845500
H	-1.50103800	6.38467900	-0.22607900
C	-1.01620700	-2.03307500	-2.95121000
C	-2.05075300	-2.95991600	-2.85102800
C	-1.86672400	-4.05163400	-2.00444700
C	-0.67891400	-4.15400500	-1.28920500
C	0.29936000	-3.15544100	-1.42393900
H	-1.11295300	-1.16875200	-3.61749500
H	-2.97012600	-2.82873300	-3.42456700
H	-2.64770300	-4.80705900	-1.88903100
H	-0.52351800	-4.97914800	-0.59181500
C	1.98974200	-2.01051000	0.08307700
C	1.53600300	-3.18496000	-0.58821000
C	2.19712200	-4.41674400	-0.43980300
C	3.32823200	-4.53928500	0.36160400
C	3.79734800	-3.40867300	1.03365000
C	3.14213800	-2.18757400	0.88682600
H	1.82539400	-5.29376200	-0.97856500
H	3.83765600	-5.50144500	0.45743400
H	4.68204600	-3.47885600	1.67389500
H	3.53918600	-1.32309300	1.42920500
C	3.55190500	1.38795600	0.02112100
C	2.74656300	0.56339800	-0.81746700
C	3.21593700	0.37622500	-2.13758500
C	4.38688100	0.96665600	-2.60708300
C	5.15599100	1.77520600	-1.76299400
C	4.73473200	1.97966000	-0.45365900
H	2.63970900	-0.26022200	-2.81797900
H	4.70927000	0.79437800	-3.63844600
H	6.07799500	2.23812000	-2.12297000
H	5.34401200	2.60652700	0.20232400
C	2.01068200	1.85971400	3.95039600
C	1.45032500	1.04366100	2.97623000
C	3.08253300	1.60131600	1.41058500
C	3.70139900	2.44715400	2.34452100
C	3.16240400	2.57420600	3.61835300
H	1.55531800	1.93293500	4.93914500
H	0.54481400	0.46183900	3.17545500
H	4.59665100	3.00832500	2.07613000
H	3.63756000	3.23183600	4.34984100
N	0.12226600	-2.12627300	-2.26477500
N	1.97235100	0.92138800	1.75543700
Ir	1.04071400	-0.20345700	-0.06543000

Ir^{III}

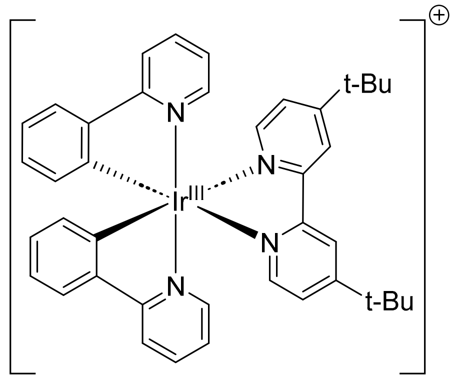

E=-1871.059723
G_{Corr}= 0.633024

C	-0.65440800	-2.60124800	0.60298300
C	-1.93302800	-0.72586800	0.16264000
C	-1.78049400	-3.39011100	0.76262700
H	0.34726400	-3.02131300	0.71419700
C	-3.10580000	-1.46651700	0.30905600
H	-1.64739500	-4.44638600	1.00044900
H	-4.06459600	-0.97153100	0.17655000
C	-0.65345600	2.60199600	-0.60076600
C	-1.93274700	0.72696200	-0.16086500
C	-1.77924400	3.39120200	-0.76069400
H	0.34838600	3.02173000	-0.71169600
C	-3.10526400	1.46797800	-0.30760400
H	-1.64579400	4.44745000	-0.99845700
H	-4.06423000	0.97323200	-0.17547100
N	-0.72388900	-1.29681600	0.31285600
N	-0.72340800	1.29754100	-0.31078000
C	-3.05462500	2.82865300	-0.61662700
C	-3.05565800	-2.82720400	0.61815800

C	-4.30792400	-3.68621000	0.79701200
C	-5.59446800	-2.87769300	0.60043400
C	-4.27517400	-4.82715000	-0.23534300
C	-4.30335700	-4.27236000	2.22024600
H	-5.68050900	-2.05793200	1.33041700
H	-5.66067900	-2.44935500	-0.41166200
H	-6.46474100	-3.53668200	0.73860100
H	-3.39493900	-5.47431800	-0.10466700
H	-5.17220800	-5.45639500	-0.12580100
H	-4.25998400	-4.43072800	-1.26278100
H	-5.20116700	-4.89158100	2.37225700
H	-3.42384800	-4.90863800	2.40024700
H	-4.30872400	-3.47242200	2.97729000
C	-4.30656200	3.68806300	-0.79588400
C	-4.30110400	4.27466400	-2.21890700
C	-5.59349700	2.87992300	-0.60020400
C	-4.27388400	4.82867600	0.23685700
H	-3.42118800	4.91053400	-2.39837000
H	-4.30658300	3.47497700	-2.97621700
H	-5.19852600	4.89440600	-2.37107400
H	-5.66047100	2.45141900	0.41177200
H	-6.46346800	3.53922800	-0.73875400
H	-5.67936100	2.06033500	-1.33040100
H	-5.17071300	5.45819100	0.12720600
H	-4.25914700	4.43192300	1.26417200
H	-3.39342300	5.47563400	0.10667200
C	0.36098700	-0.29676700	-2.95094000
C	0.46436500	-0.82435100	-4.22738400
C	1.37399100	-1.85811400	-4.44782700
C	2.14327900	-2.32204900	-3.39006300
C	1.99769000	-1.75014100	-2.12265800
H	-0.33230700	0.51537800	-2.72786000
H	-0.15869600	-0.42701000	-5.02900900
H	1.48416700	-2.29861200	-5.44077900
H	2.86255800	-3.12684000	-3.54206200
C	2.44626300	-1.38406600	0.24504600
C	2.75169700	-2.13221800	-0.91879000
C	3.70093200	-3.16326100	-0.89651700
C	4.36893400	-3.46636500	0.28561900
C	4.08509700	-2.73629800	1.44373400
C	3.13991300	-1.71046300	1.42179000
H	3.92297400	-3.73606100	-1.80003500
H	5.10886200	-4.26965900	0.30599200
H	4.60899700	-2.97064500	2.37462400
H	2.93916100	-1.15834200	2.34452100
C	2.75460100	2.13062200	0.91672300
C	2.44702800	1.38260500	-0.24663100
C	3.13950400	1.70823000	-1.42430400
C	4.08542600	2.73332000	-1.44761300
C	4.37124300	3.46339600	-0.28997700
C	3.70458100	3.16096600	0.89307300
H	2.93717800	1.15582000	-2.34648900
H	4.60836700	2.96710200	-2.37918500
H	5.11172400	4.26615400	-0.31144000
H	3.92825500	3.73378100	1.79617000
C	0.47050700	0.82487100	4.22839700
C	0.36493900	0.29743000	2.95208200
C	2.00196500	1.74919700	2.12163500
C	2.14985800	2.32088700	3.38887400
C	1.38151800	1.85767500	4.44765000
H	-0.15194400	0.42821800	5.03083500
H	-0.32952500	-0.51387900	2.72968100
H	2.87018900	3.12489900	3.54000200
H	1.49347800	2.29801700	5.44047400
N	1.10499500	-0.74634000	-1.92928500
N	1.10806200	0.74624800	1.92946700
Ir	1.00546200	-0.00010000	0.00018300

0

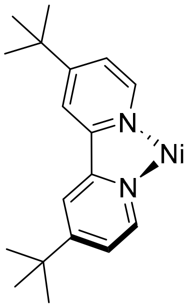

E=-2317.835956

G_{Corr}= 0.33395

C	-2.58107200	1.85396200	0.30898300
C	-0.69966300	0.45265900	0.09197500
C	-3.44254900	0.80289000	0.16730900
H	-2.95808500	2.87251200	0.44684000
C	-1.56820700	-0.67362600	-0.01532000
H	-4.51654600	0.99654800	0.19599400
H	-1.10777800	-1.64746200	-0.18771200
C	2.58121100	1.85332800	-0.31125200
C	0.69945400	0.45250800	-0.09326800
C	3.44241000	0.80219600	-0.16876300
H	2.95844600	2.87168400	-0.44994900
C	1.56778600	-0.67382200	0.01494200
H	4.51646800	0.99556000	-0.19761100
H	1.10718500	-1.64744900	0.18807000
Ni	0.00098300	3.03699000	0.00118400
N	-1.22579600	1.71658200	0.37394000
N	1.22577200	1.71624900	-0.37611600
C	2.93294200	-0.53877800	-0.03452400
C	-2.93333200	-0.53824800	0.03406800
C	-3.91129400	-1.70992600	-0.07973900
C	-3.18690800	-3.05343000	-0.21710800
C	-4.79012600	-1.75689900	1.18363600
C	-4.80743400	-1.51000200	-1.31555900
H	-2.56783500	-3.09450800	-1.12689700
H	-2.53743200	-3.25850700	0.64831500
H	-3.92348500	-3.86950900	-0.28097900
H	-5.36836400	-0.82956000	1.31579100
H	-5.50836200	-2.59057400	1.12169100
H	-4.17528900	-1.90446600	2.08599000
H	-5.52130600	-2.34418700	-1.41216500
H	-5.39172100	-0.57916400	-1.25330600
H	-4.20447200	-1.47063100	-2.23694600
C	3.91071000	-1.71051900	0.08013900
C	4.80677500	-1.50991500	1.31589900
C	3.18611500	-3.05382800	0.21830400
C	4.78965900	-1.75842200	-1.18312200
H	5.39114200	-0.57915900	1.25311300
H	4.20374800	-1.46992100	2.23721600
H	5.52057500	-2.34409800	1.41307600
H	2.53666900	-3.25935600	-0.64703600
H	3.92256900	-3.86997300	0.28274300
H	2.56696700	-3.09424600	1.12807400
H	5.50782800	-2.59211000	-1.12056900
H	4.17488700	-1.90652900	-2.08543300
H	5.36797700	-0.83121000	-1.31584200

A

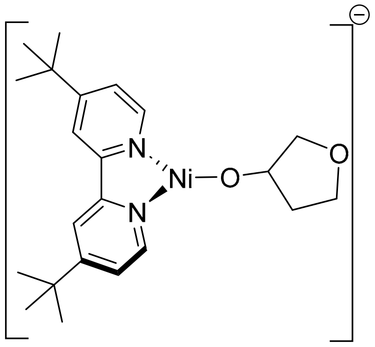

E=-2624.990609
G_{Corr}= 0.432849

C	-1.29046300	-2.66035100	-0.56099000
C	-1.26388800	-0.30553700	-0.22160500
C	-2.63633500	-2.74992500	-0.31488800
H	-0.71334800	-3.55760100	-0.81812100
C	-2.67820400	-0.36994900	0.09136600
H	-3.12906800	-3.72141500	-0.37173800
H	-3.17972400	0.56739500	0.33820400
C	1.77904300	1.49936500	-0.80877100
C	-0.45025700	0.80029300	-0.29802000
C	1.49995900	2.80913200	-0.52648400
H	2.77538900	1.18821100	-1.13812900
C	-0.76346900	2.17786500	0.02663500
H	2.28269300	3.55885100	-0.65021200
H	-1.77420300	2.38596500	0.38231300
Ni	1.22985300	-1.27966300	-0.83930500
N	-0.57905200	-1.52001300	-0.47894800
N	0.86802600	0.50475900	-0.74446900
C	0.15915700	3.17518700	-0.08219800
C	-3.36886800	-1.54561400	0.05435400
C	-4.86391300	-1.65746000	0.36776000
C	-5.48751900	-0.30533600	0.73086400
C	-5.60168600	-2.21529200	-0.86340600
C	-5.06390000	-2.61704700	1.55532000
H	-5.01660400	0.13610900	1.62310300
H	-5.40178500	0.41960100	-0.09365400
H	-6.55935200	-0.43370000	0.94994100
H	-5.23075800	-3.21163100	-1.14824100
H	-6.68093200	-2.30804500	-0.65842300
H	-5.47767700	-1.54800400	-1.73165100
H	-6.13534000	-2.71929300	1.79398600
H	-4.67352100	-3.62339700	1.34014300
H	-4.55050800	-2.24110600	2.45508800
C	-0.13378100	4.64021400	0.25270700
C	0.11382100	5.50585500	-0.99666200
C	-1.58082000	4.85663200	0.70877500
C	0.80488800	5.10284900	1.38230000
H	1.15308700	5.42866900	-1.35077400
H	-0.54804900	5.20131300	-1.82348300
H	-0.08464800	6.56784400	-0.77709500
H	-1.81228500	4.28573200	1.62156400
H	-1.74827100	5.92212400	0.93238500
H	-2.30296700	4.56304200	-0.06914200
H	0.61599700	6.15930800	1.63463200
H	0.64840500	4.50325900	2.29363500
H	1.86429600	5.01308500	1.09753100
O	3.01343200	-1.37197800	-1.10990000
C	3.84609300	-1.27905600	-0.02417600
C	3.43097700	-2.03926700	1.24599200
C	5.21958000	-1.91492300	-0.27444300
H	4.01422900	-0.21422500	0.26692600
C	4.75217800	-2.18204600	1.98685800
H	3.03159400	-3.02201600	0.93979300
H	2.65963700	-1.51856000	1.83217000
H	5.90832400	-1.25386700	-0.82588000
H	5.07037400	-2.83426900	-0.87653000
H	4.92587200	-1.32266000	2.66150700
H	4.80813500	-3.09819800	2.59826300
O	5.76991400	-2.21411000	0.99097600

B

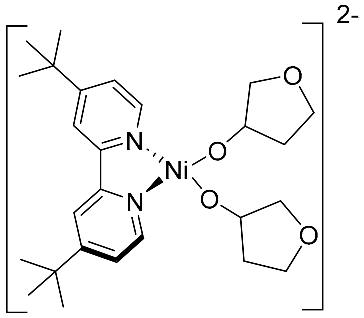

E=-2932.134451
G_{Corr}= 0.536191

C	0.01402200	2.47389800	-0.54821600
C	1.51240800	0.67985200	-0.13904200
C	1.03346500	3.38722100	-0.72032100
H	-1.04078500	2.75534900	-0.64228800
C	2.61645000	1.61724200	-0.29030300
H	0.79327300	4.42497000	-0.95788200
H	3.62760600	1.22366300	-0.17000300
C	0.26501800	-2.61968000	0.42509200
C	1.57921800	-0.66975400	0.10208700
C	1.37008500	-3.43149000	0.57758000
H	-0.75767700	-3.00944200	0.48505800
C	2.77064400	-1.49191800	0.26025300
H	1.23471900	-4.49858700	0.76239300
H	3.73823800	-0.99226100	0.18393000
Ni	-1.16499000	-0.12605100	-0.01012400
N	0.18543300	1.18055700	-0.25560800
N	0.30898300	-1.30266400	0.19945800
C	2.69499000	-2.83427800	0.49085600
C	2.41010400	2.93555700	-0.57346300
C	3.54677900	3.94991100	-0.73996100
C	4.93110100	3.31705400	-0.55872600
C	3.47965100	4.56478300	-2.15025600
C	3.38787600	5.06942200	0.30539100
H	5.05349700	2.88169800	0.44535500
H	5.11750300	2.52284400	-1.29855200
H	5.71413000	4.08134900	-0.68727800
H	2.52316500	5.07917300	-2.32838600
H	4.28650000	5.30308900	-2.29114800
H	3.59319500	3.78774000	-2.92358200
H	4.19089200	5.81770900	0.19948300
H	2.42656600	5.59476100	0.19963300
H	3.43775900	4.66161400	1.32812600
C	3.92680400	-3.72997100	0.66170300
C	3.91191400	-4.83001100	-0.41579000
C	5.24181800	-2.95257200	0.53452600
C	3.88876800	-4.38747700	2.05366900
H	3.01014400	-5.45734400	-0.34873500
H	3.94194200	-4.39059600	-1.42614400
H	4.78743200	-5.49165500	-0.30808700
H	5.32687700	-2.16457900	1.29912700
H	6.09664000	-3.63531400	0.66474200
H	5.34272300	-2.47879900	-0.45450400
H	4.76152400	-5.04614100	2.19669800
H	3.90666000	-3.62459000	2.84888100
H	2.98385700	-4.99803600	2.19376300
O	-2.38741300	-1.52604900	0.27704800
O	-2.53661700	1.14093400	-0.23845600

C	-3.45741700	-1.62265700	-0.55495800
C	-3.18314100	-1.56057700	-2.06902100
C	-4.15598000	-2.98874500	-0.48336500
H	-4.23793800	-0.84718100	-0.35008700
C	-4.42211400	-2.21818400	-2.65775500
H	-2.27183400	-2.14787100	-2.27651900
H	-3.02689400	-0.52983400	-2.41523100
H	-4.83314700	-3.08767500	0.38203700
H	-3.38116100	-3.77956600	-0.40213000
H	-5.20805500	-1.46978200	-2.87509100
H	-4.22217900	-2.76372700	-3.59641700
C	-3.27091200	1.37137100	0.88287400
C	-2.61339000	2.19894500	2.00923100
C	-4.51046900	2.24090500	0.62353900
H	-3.62402000	0.42131300	1.35538100
C	-3.82179800	2.66963000	2.80015800
H	-2.07804800	3.05158100	1.55847700
H	-1.89651700	1.62094900	2.61166200
H	-5.36342300	1.66537300	0.22415300
H	-4.24433200	3.01733300	-0.12298200
H	-4.11466800	1.92094700	3.56223500
H	-3.66018800	3.62610100	3.32558400
O	-4.90484000	-3.13028200	-1.67427500
O	-4.87744700	2.82899500	1.85695200

C

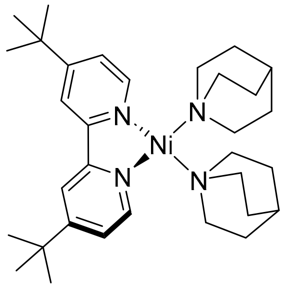

E=-2976.269756

G_{Corr}= 0.712496

C	0.32263900	-2.41265900	-1.03712400
C	1.66522500	-0.64599600	-0.25214800
C	1.40274900	-3.22239100	-1.25270100
H	-0.68467800	-2.76828500	-1.27982100
C	2.82387400	-1.47222000	-0.44349000
H	1.24483600	-4.21472300	-1.67934000
H	3.79094100	-1.05899800	-0.15302400
C	0.28414300	2.41126000	1.02306200
C	1.65489000	0.66308600	0.24624100
C	1.35186400	3.23601200	1.24421500
H	-0.72929900	2.75309200	1.26025800
C	2.80099300	1.50560600	0.44222900
H	1.17816400	4.22593200	1.67031100
H	3.77508900	1.10592700	0.15636900
Ni	-1.04378200	-0.01056500	-0.01218100
N	0.39447200	-1.13720700	-0.57513600
N	0.37597900	1.13728700	0.56114700
C	2.68838600	2.77545600	0.94038400
C	2.73141100	-2.74329000	-0.94257000
C	3.93360200	-3.66428700	-1.16365000
C	5.25495300	-2.99552000	-0.76943100
C	3.76198400	-4.93973300	-0.31852400
C	4.00931700	-4.04940900	-2.65263500
H	5.44410400	-2.08421200	-1.35814000
H	5.27351000	-2.72251400	0.29726400
H	6.09292200	-3.68721600	-0.94944800
H	2.84730100	-5.48984600	-0.58793800
H	4.61579000	-5.62083900	-0.46797600
H	3.70607200	-4.69607600	0.75471800
H	4.86557800	-4.71917500	-2.83524600
H	3.10113900	-4.57346100	-2.98780100
H	4.13680300	-3.15554000	-3.28428900
C	3.87665700	3.71309000	1.16688200
C	3.94099500	4.09797000	2.65644100
C	5.20887800	3.06338100	0.77746100
C	3.69045700	4.98670900	0.32205800
H	3.02412100	4.60870100	2.98850600
H	4.07877400	3.20550800	3.28792600
H	4.78687000	4.77976000	2.84297600
H	5.23535100	2.79104600	-0.28924000
H	6.03618700	3.76691400	0.96086400
H	5.40885300	2.15467800	1.36660900
H	4.53415700	5.67947300	0.47523900
H	3.64195000	4.74318300	-0.75156700
H	2.76721100	5.52377000	0.58855100
C	-3.46485300	1.87366100	-0.37862600
H	-4.11791500	1.05284300	-0.05309500
H	-3.09188300	2.37782300	0.52417100
C	-2.80019800	0.40310500	-2.17684500
H	-1.91923200	0.09404500	-2.75739600
H	-3.23969400	-0.50343200	-1.74749200
C	-1.50013900	2.35220900	-1.68029700
H	-0.56119700	1.91408300	-2.04461000
H	-1.24088700	3.05651700	-0.88114300
N	-2.30953200	1.26589500	-1.07251900
C	-4.23058000	2.85114400	-1.29792000
H	-4.05519100	3.89455200	-0.99200300
H	-5.31370700	2.66940900	-1.22744000
C	-2.27203000	3.06811700	-2.81038300
H	-1.85708500	2.80411700	-3.79590900
C	-3.82256700	1.14809700	-3.06315100
H	-4.84326500	0.77807300	-2.87655800
H	-3.60170500	0.97196800	-4.12670700
C	-3.74130900	2.64220200	-2.73508800
H	-4.35120000	3.22703300	-3.43882700
H	-2.17902900	4.15924500	-2.70055900
C	-2.75570400	-0.43745300	2.18258000
H	-1.86233000	-0.12875800	2.74402800
H	-3.20442700	0.46955000	1.76364900
C	-1.46881400	-2.38735300	1.65516000
H	-1.22641400	-3.08972900	0.84897800
H	-0.52220900	-1.95097200	2.00121800
C	-3.45962800	-1.90410400	0.39637500
H	-3.10610300	-2.40838700	-0.51413900
H	-4.11784700	-1.08200300	0.08482200
N	-2.28924500	-1.29902900	1.06681800
C	-3.75862300	-1.18313600	3.09036000
H	-4.78273700	-0.81097700	2.92835400
H	-3.51311500	-1.01024000	4.14904600
C	-4.20835600	-2.88107400	1.33025900
H	-4.04290200	-3.92441700	1.01863400
C	-2.21787600	-3.10578000	2.79903600
H	-1.78115500	-2.84617300	3.77628000
H	-2.12974500	-4.19673300	2.68349100
C	-3.68754300	-2.67661200	2.75689600
H	-4.28318000	-3.26188800	3.47238000
H	-5.29221500	-2.69597900	1.28361300

D

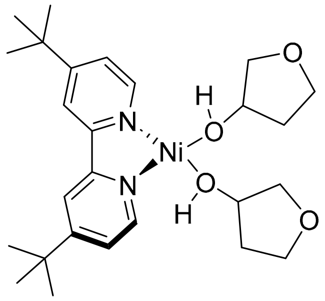

E=-2933.102844
G_{Corr}= 0.56582

C	0.31645300	2.37267300	0.61158700
C	-1.30861100	0.70136500	0.19834800
C	-0.63084200	3.33832700	0.78952000
H	1.37156000	2.64741700	0.70683100
C	-2.33416500	1.69848000	0.38072800
H	-0.31043200	4.35110400	1.03893300
H	-3.36768500	1.37960200	0.23745700
C	-0.35339500	-2.60668600	-0.79616600
C	-1.48823800	-0.62333600	-0.15981200
C	-1.51179400	-3.32775000	-0.83587000
H	0.60207200	-3.08067600	-1.03961900
C	-2.72810900	-1.35905700	-0.19211300
H	-1.47053000	-4.37818800	-1.12846600
H	-3.63347200	-0.82208300	0.09452900
Ni	1.23141200	-0.28721600	-0.14581400
N	0.04751200	1.06103500	0.37382000
N	-0.29191300	-1.27618800	-0.52231700
C	-2.77540500	-2.68112300	-0.52641200
C	-2.03882300	2.99631500	0.67806200
C	-3.08972500	4.09155200	0.86820200
C	-4.51729800	3.55887900	0.70540600
C	-2.95047000	4.68632900	2.28170600
C	-2.86184400	5.20213700	-0.17367300
H	-4.68824000	3.14694800	-0.30146800
H	-4.74571800	2.77065600	1.43982400
H	-5.24120400	4.37486600	0.85740800
H	-1.95356600	5.12291300	2.44702800
H	-3.69334500	5.48532300	2.43916400
H	-3.11361100	3.91465300	3.05121300
H	-3.60539300	6.00650700	-0.05015900
H	-1.86316000	5.65522300	-0.07808300
H	-2.95687000	4.80617600	-1.19764000
C	-4.06458600	-3.50469500	-0.56626200
C	-3.95568900	-4.67145000	0.43254500
C	-5.29730200	-2.66968300	-0.20341100
C	-4.26075300	-4.06893800	-1.98552600
H	-3.11172100	-5.33723000	0.19560000
H	-3.81401400	-4.29804000	1.45960100
H	-4.87428900	-5.28058000	0.41659200
H	-5.43897100	-1.82678900	-0.89786500
H	-6.20169100	-3.29631800	-0.25215400
H	-5.23024100	-2.26296300	0.81774800
H	-5.18088600	-4.67378500	-2.03626600
H	-4.34824100	-3.25554200	-2.72375200
H	-3.42257000	-4.71379000	-2.29088200
O	2.47748100	-1.84231600	-0.12096700
O	2.89405000	0.80757700	-0.28567100
C	2.48802400	-2.61587400	1.09484900
C	3.41061600	-3.82420100	0.94705000
C	3.18500000	-1.83428100	2.19617900
H	1.44348800	-2.86822300	1.30928700
C	4.76526400	-3.29606800	1.47210000
H	3.45033900	-4.16354800	-0.09673300
H	3.03839300	-4.65522000	1.56139300
H	2.97687600	-2.29707400	3.18015100

H	2.89692400	-0.77436000	2.23508000
H	5.10009300	-3.87691200	2.34971600
H	5.56556200	-3.31994000	0.71907200
C	3.32975700	1.26317600	-1.58436200
C	2.28583300	2.14828500	-2.26315500
C	4.47444900	2.24151400	-1.38916900
H	3.59121100	0.36268900	-2.15305800
C	2.67993500	3.56860200	-1.80279700
H	1.26924600	1.85590000	-1.96788900
H	2.36744100	2.04171700	-3.35345500
H	5.00440600	2.39980800	-2.34757800
H	5.20044000	1.92612900	-0.62668200
H	2.94743200	4.20272400	-2.66644000
H	1.88963700	4.07716400	-1.23318200
O	4.54570300	-1.93904000	1.83835000
O	3.80876400	3.40654500	-0.95046000
H	3.37883400	-1.50580200	-0.25324100
H	2.98548600	1.53857900	0.34494400

E

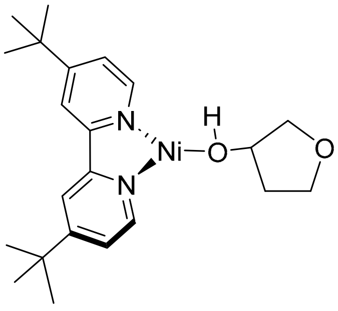

E=-2625.477104
G_{Corr}= 0.447152

C	0.44159300	-2.74856800	-0.55128500
C	1.06689900	-0.51181200	-0.19417700
C	1.69594400	-3.19863000	-0.24398700
H	-0.35784900	-3.44795800	-0.81597600
C	2.40524200	-0.93212900	0.05310500
H	1.88539700	-4.27360700	-0.25088100
H	3.13488200	-0.16678600	0.32096300
C	-1.43147700	2.06076500	0.06391300
C	0.54504500	0.79259700	-0.02896200
C	-0.73544200	3.23628900	0.10649900
H	-2.52554200	2.06007300	0.07487300
C	1.30608000	1.99865300	-0.01153100
H	-1.29096500	4.17323200	0.18089500
H	2.38963500	1.91199500	-0.10383500
Ni	-1.57267100	-0.70785700	-0.74482700
N	0.11934700	-1.42860500	-0.63701300
N	-0.84644100	0.83012500	0.05838200
C	0.70301700	3.22898600	0.07495200
C	2.75381300	-2.26107800	0.02319400
C	4.17080400	-2.77563200	0.28712200
C	5.15593100	-1.63422600	0.56088300
C	4.15500200	-3.71169700	1.50917600
C	4.65981800	-3.55678400	-0.94631500
H	5.22078500	-0.93707900	-0.28915000
H	4.87661300	-1.05709400	1.45615400
H	6.16339400	-2.04443300	0.73209600
H	3.49668000	-4.57992200	1.35278500
H	5.16802800	-4.09523800	1.71255300
H	3.80625300	-3.17945800	2.40867800
H	5.67824900	-3.94293700	-0.77786700
H	4.01083200	-4.41725300	-1.17042600
H	4.68479000	-2.90986400	-1.83788300
C	1.46756100	4.55407000	0.10574000
C	1.11871200	5.30535400	1.40366300

C	2.98483500	4.34448700	0.05892800
C	1.05383500	5.41060500	-1.10467200
H	0.04202200	5.52297700	1.47529900
H	1.40526500	4.71610300	2.28947100
H	1.65564900	6.26685400	1.44689600
H	3.30079600	3.83975500	-0.86739300
H	3.49736400	5.31838400	0.09702400
H	3.34168500	3.74735300	0.91271000
H	1.59212500	6.37236600	-1.09766000
H	1.28951900	4.89610000	-2.05006500
H	-0.02434000	5.63278600	-1.09970300
O	-3.41107900	-0.14046600	-1.22421900
C	-4.47619800	-0.64914900	-0.42064900
C	-4.47748500	-2.16367000	-0.26192600
C	-4.31923000	-0.18002000	1.02536000
H	-5.42659300	-0.29430800	-0.84760000
C	-5.23982100	-2.31908300	1.04442600
H	-3.43291900	-2.50376500	-0.14711300
H	-4.93535700	-2.69811800	-1.10498900
H	-4.80392300	0.79148400	1.21025300
H	-3.23962500	-0.08117100	1.25017900
H	-6.33063500	-2.34947100	0.86469500
H	-4.96276900	-3.23035000	1.59560400
O	-4.90923200	-1.17806400	1.82699900
H	-3.64940100	-0.21901200	-2.15800300

G

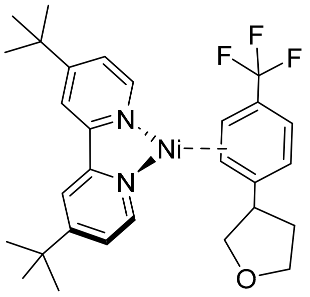

E=-3118.34295
G_{Corr}= 0.521109

C	0.75838600	-2.56703200	-0.98672200
C	1.82557000	-0.63378500	-0.32460500
C	1.96277000	-3.24220400	-1.11108400
H	-0.18928500	-3.06660400	-1.19964500
C	3.07244700	-1.24776000	-0.43200500
H	1.94658200	-4.28660200	-1.42690600
H	3.96796900	-0.67455600	-0.20085300
C	0.07596500	2.42035100	0.54233100
C	1.63003000	0.77227400	0.12058600
C	1.05463200	3.28902600	0.99934300
H	-0.97432200	2.71709700	0.53153900
C	2.66667300	1.59444200	0.55972600
H	0.75379600	4.27901900	1.34576900
H	3.68523800	1.21168200	0.55712600
Ni	-0.91626700	-0.16362400	-0.52790000
N	0.68387200	-1.28859600	-0.60283500
N	0.34989500	1.18893100	0.09849000
C	2.39823900	2.88968500	1.01043500
C	3.16886100	-2.58275600	-0.83422200
C	4.50422700	-3.31699300	-0.97116700
C	5.69524600	-2.41339800	-0.63492200
C	4.50740600	-4.51894400	-0.00979800
C	4.65265600	-3.81189500	-2.42087900
H	5.75281500	-1.54282100	-1.30659100
H	5.65394800	-2.04779700	0.40276000
H	6.63047400	-2.98218900	-0.74853800
H	3.69934000	-5.22984700	-0.23921400
H	5.46288100	-5.06092100	-0.08938200
H	4.38778400	-4.19013900	1.03456800
H	5.61232400	-4.33867400	-2.54120500
H	3.85057700	-4.51133200	-2.70068000
H	4.63524700	-2.96935900	-3.13010200
C	3.48771500	3.84147000	1.50804400
C	3.18812900	4.21519500	2.97078500
C	4.88102300	3.20790100	1.43590200
C	3.47483500	5.10997800	0.63643200
H	2.21676400	4.72198900	3.07426600
H	3.17766700	3.32067200	3.61334100
H	3.96410800	4.89844300	3.35026400
H	5.15703700	2.93859700	0.40459200
H	5.62980800	3.92617100	1.80262300
H	4.95467900	2.30454500	2.06091900
H	4.25951300	5.80500000	0.97432600
H	3.66749500	4.86629800	-0.42028500
H	2.51200800	5.63968200	0.69564400
C	-2.96359700	-1.93730700	0.04858300
C	-2.55576100	-1.03352200	-0.99172100
C	-2.71100200	0.42609400	-0.76326300
C	-3.32102200	0.81447100	0.49837400
C	-3.63602500	-0.07879800	1.47910600
C	-3.44398100	-1.49135700	1.25114600
H	-2.89561500	-3.01315900	-0.13698300
H	-2.58666900	-1.43185800	-2.01627200
H	-3.53707500	1.87510200	0.66877000
H	-4.06055400	0.26478800	2.42588000
F	-3.63614100	-3.72433500	2.01360200
F	-5.07716800	-2.30261500	2.75214500
F	-3.05129500	-2.23544100	3.45598100
C	-3.79634800	-2.43548300	2.34609600
C	-2.94222300	1.31093200	-1.97680700
C	-4.41215200	1.52697200	-2.35320800
C	-2.46078100	2.76257300	-1.90562900
H	-2.44296000	0.84116500	-2.84179400
H	-5.02389200	1.66887700	-1.43984500
H	-4.83689400	0.67574100	-2.91095500
H	-1.37165600	2.85830900	-2.01887000
H	-2.74719800	3.21905800	-0.94395000
C	-3.23414700	3.40395100	-3.05118500
H	-2.67178900	3.32130200	-4.00079400
H	-3.44770100	4.47233100	-2.88261700
O	-4.45731500	2.68952000	-3.15860900

F

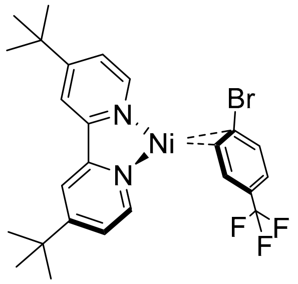

E=-5460.630743
G_{Corr}= 0.416551

C	0.72513600	-2.59590100	0.17189000
C	1.77979800	-0.54874000	0.03776400
C	1.89945300	-3.22603600	0.56994300
H	-0.20094400	-3.16201400	0.04688300
C	2.98695600	-1.11108400	0.43117700
H	1.87080600	-4.29771500	0.76257900
H	3.86882000	-0.47669300	0.52209500

C	0.07245900	2.49883400	-0.93020800
C	1.59601300	0.89324500	-0.28003700
C	1.03876600	3.49856800	-0.88856200
H	-0.95816200	2.72869000	-1.20944700
C	2.61125300	1.83847700	-0.22001600
H	0.74175100	4.51517200	-1.14290400
H	3.61571700	1.52944200	0.06907100
Ni	-0.91470600	-0.27575500	-0.61637100
N	0.65946500	-1.29037300	-0.08572000
N	0.33786900	1.22665900	-0.63570300
C	2.35074500	3.18235900	-0.52680200
C	3.07409300	-2.48274100	0.71210800
C	4.40886200	-3.09228300	1.14492800
C	5.43888100	-2.87837800	0.02120900
C	4.88586600	-2.38787700	2.42760000
C	4.28757600	-4.59406200	1.42330600
H	5.11045800	-3.36014700	-0.91315700
H	5.60686600	-1.81055800	-0.18529400
H	6.40595900	-3.31887600	0.31034100
H	4.15507200	-2.51213500	3.24218500
H	5.84264100	-2.82092200	2.75901400
H	5.04423900	-1.31012200	2.27174100
H	5.26623400	-4.98908900	1.73534800
H	3.57087300	-4.80477200	2.23214200
H	3.97388200	-5.15357700	0.52849600
C	3.47689200	4.21525500	-0.45417500
C	4.03625500	4.24093400	0.97956900
C	4.58862600	3.80634000	-1.43695300
C	2.98757800	5.62083900	-0.81786800
H	3.25300900	4.51176100	1.70497500
H	4.45500200	3.26723400	1.27558200
H	4.84255700	4.98738100	1.05374200
H	4.20829400	3.76442100	-2.46973000
H	5.40695600	4.54261400	-1.40452600
H	5.01468200	2.82236500	-1.18894000
H	3.82685600	6.32965900	-0.75185400
H	2.59507200	5.66594500	-1.84552900
H	2.20142900	5.97191600	-0.13159100
C	-3.70237100	-0.62069500	1.39484800
C	-3.03758900	-1.55878500	0.66407700
C	-2.47821300	-1.25558800	-0.64039500
C	-2.72053400	0.10175300	-1.18040900
C	-3.52760600	1.00449200	-0.40971500
C	-3.96792800	0.68459200	0.84593700
H	-4.06137800	-0.86809300	2.39736500
H	-2.90220200	-2.56412900	1.07354000
H	-2.73640200	0.25751500	-2.26676100
H	-3.80316800	1.96584800	-0.85192200
Br	-2.56607300	-2.79093600	-1.89721300
F	-4.18859400	1.89328700	2.85818500
F	-5.99729500	1.18144800	1.94928700
F	-4.93204400	2.83305500	1.06768600
C	-4.76272100	1.64459600	1.66260600

2

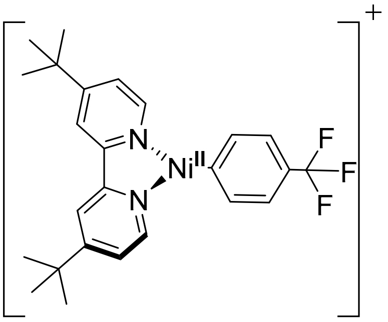

E = -2886.391503

G_{Corr}= 0.420737

C	-1.73897200	-2.91487600	-0.23079500
C	-2.10939600	-0.63695900	-0.06295800
C	-3.09628800	-3.18519600	-0.09951000
H	-1.00912300	-3.71889500	-0.35258000
C	-3.47353000	-0.83346000	0.08148000
H	-3.42082500	-4.22429700	-0.12454700
H	-4.13049800	0.02641500	0.21161100
C	0.61077700	1.75589700	-0.25235400
C	-1.43196900	0.67924400	-0.08385200
C	-0.00050300	3.00248800	-0.21146300
H	1.69265000	1.67377900	-0.34455700
C	-2.09691400	1.89277100	-0.03774800
H	0.63682700	3.88320000	-0.27393200
H	-3.18403000	1.89586200	0.03533700
Ni	0.60885600	-1.13332100	-0.23130100
N	-1.26257100	-1.67208900	-0.21278100
N	-0.07978600	0.61402400	-0.17936000
C	-1.38770400	3.10185900	-0.09447900
C	-4.00316000	-2.13399600	0.06599700
C	-5.50940800	-2.34290000	0.22592100
C	-5.95270300	-1.74176500	1.57206100
C	-6.23230300	-1.62513900	-0.92836600
C	-5.88725100	-3.82730600	0.19863000
H	-5.43389300	-2.22865800	2.41265400
H	-5.75515700	-0.66055800	1.62685400
H	-7.03523500	-1.89057600	1.70716800
H	-5.91443600	-2.02511200	-1.90395300
H	-7.31934700	-1.77437600	-0.83642700
H	-6.04272900	-0.54115400	-0.92388400
H	-6.97603400	-3.92893700	0.32103000
H	-5.61470500	-4.30319100	-0.75603300
H	-5.40727100	-4.38809700	1.01547900
C	-2.13488800	4.43270700	-0.03044000
C	-3.13688400	4.49830200	-1.19716500
C	-2.89008300	4.50615300	1.30923200
C	-1.18057400	5.62696800	-0.12897200
H	-2.62125500	4.41920300	-2.16700500
H	-3.88992500	3.69782200	-1.14178900
H	-3.67178300	5.46031800	-1.17260700
H	-2.19359900	4.44424700	2.15999400
H	-3.43048700	5.46291900	1.37925600
H	-3.62866500	3.69650100	1.41039400
H	-1.75921700	6.56156600	-0.07994700
H	-0.45475800	5.64203000	0.69867300
H	-0.62402600	5.62995600	-1.07890000
C	2.43220800	-0.74841600	-0.12416600
C	3.24646600	-1.28488900	-1.13082000
C	3.05661900	-0.15365300	0.98437800
C	4.63952300	-1.23123100	-1.04366300
H	2.79969900	-1.75614000	-2.01281400
C	4.44368100	-0.09458500	1.08492100
H	2.45319500	0.28072400	1.78744500
C	5.23647100	-0.63414500	0.06546500
H	5.25512400	-1.65062100	-1.84170800
H	4.91062300	0.37099900	1.95676500
C	6.73144100	-0.55716300	0.19574800
F	7.15551800	0.70820700	0.34441900
F	7.17459300	-1.22921100	1.27107400
F	7.37094000	-1.05896500	-0.86583300

2’

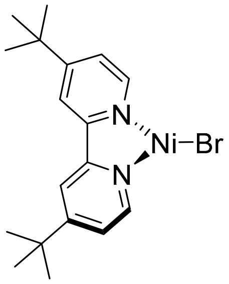

E=-4892.064635
G_{Corr}= 0.331608

C	0.95404600	2.61510300	0.03566700
C	-0.39563000	0.74530700	0.00395300
C	-0.14466300	3.46695400	0.02864500
H	1.97233200	3.01192100	0.05143800
C	-1.53890000	1.53515200	-0.00972300
H	0.03424600	4.54138700	0.04082400
H	-2.51997200	1.06007300	-0.03454700
C	0.92755800	-2.62517300	-0.03717500
C	-0.40318800	-0.74190200	-0.00382600
C	-0.17963600	-3.46588800	-0.02989000
H	1.94176700	-3.03227300	-0.05372500
C	-1.55443200	-1.52019600	0.01043700
H	-0.01166700	-4.54208100	-0.04278200
H	-2.53068800	-1.03529700	0.03625700
Ni	2.31829900	-0.01223400	-0.00032600
N	0.83937700	1.28674000	0.02378900
N	0.82631000	-1.29572300	-0.02465900
C	-1.46665300	-2.91994100	-0.00408500
C	-1.43711300	2.93393400	0.00404500
C	-2.70304000	3.79384400	-0.00935300
C	-3.50210300	3.48193800	-1.28730600
C	-3.55146500	3.45075400	1.22821200
C	-2.37829200	5.29112200	0.01590300
H	-2.90987400	3.70539400	-2.18863700
H	-3.80883400	2.42597200	-1.33263500
H	-4.41545700	4.09660500	-1.31840400
H	-2.99411200	3.64853900	2.15736200
H	-4.46397200	4.06730100	1.24036600
H	-3.86234800	2.39505200	1.23393400
H	-3.31447000	5.86973300	0.00651500
H	-1.82143500	5.57511400	0.92232400
H	-1.78933400	5.59837400	-0.86215600
C	-2.74086100	-3.76754100	0.00963400
C	-3.53631200	-3.44852900	1.28807400
C	-3.58648500	-3.41596700	-1.22748700
C	-2.43036900	-5.26783100	-0.01628300
H	-2.94562600	-3.67748500	2.18903700
H	-3.83346600	-2.38982200	1.33375200
H	-4.45518300	-4.05486900	1.31960900
H	-3.03126500	-3.61844600	-2.15690500
H	-4.50464400	-4.02407900	-1.23965300
H	-3.88764400	-2.35743900	-1.23259300
H	-3.37197000	-5.83755700	-0.00660300
H	-1.87669600	-5.55677900	-0.92309200
H	-1.84389700	-5.58095500	0.86137500
Br	4.61935000	-0.02085200	0.00050000

TS_{1-2}

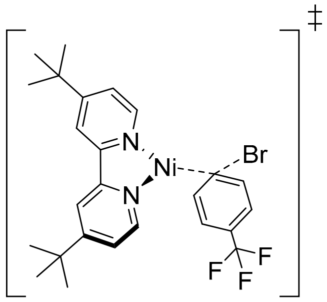

E=-5460.615071
G_{Corr}= 0.416046

C	0.69796400	-2.45410100	0.09885400
C	1.92731800	-0.49245200	0.10066100
C	1.82143300	-3.20118700	0.43875000
H	-0.27352500	-2.92171700	-0.09391400
C	3.08305400	-1.17881500	0.44029700
H	1.70694100	-4.27876000	0.54878300
H	4.01616300	-0.62872300	0.56155100
C	0.44361900	2.71418300	-0.68577300
C	1.85974600	0.97152400	-0.14088700
C	1.48701900	3.63239000	-0.64393000
H	-0.57525500	3.02979200	-0.92121100
C	2.94663600	1.83147200	-0.08098600
H	1.26398500	4.67727100	-0.85484900
H	3.93206500	1.43433200	0.16329400
Ni	-0.74171400	0.01390800	-0.40117600
N	0.74724400	-1.13131600	-0.05461900
N	0.62358200	1.41708300	-0.44289600
C	2.78052500	3.20158900	-0.33605900
C	3.05303100	-2.56987800	0.62703200
C	4.33143700	-3.31604000	1.01168800
C	5.38832000	-3.09000600	-0.08424500
C	4.84473000	-2.75895400	2.35173500
C	4.09104300	-4.82163800	1.16273200
H	5.03391300	-3.46202500	-1.05837600
H	5.64463100	-2.02577500	-0.19675200
H	6.31306200	-3.63061100	0.17125800
H	4.09723800	-2.89517800	3.14901900
H	5.76228400	-3.28971400	2.65023800
H	5.08504100	-1.68687000	2.28770600
H	5.03379300	-5.31691100	1.44026300
H	3.35504900	-5.04254100	1.95118100
H	3.74012600	-5.27741700	0.22404100
C	3.98553900	4.14104600	-0.26919100
C	4.57928000	4.08489500	1.14987900
C	5.03633200	3.67248100	-1.29168600
C	3.60007100	5.58954400	-0.58641100
H	3.83835800	4.40014300	1.90127000
H	4.92474900	3.07371800	1.41297000
H	5.44477200	4.76218300	1.21947500
H	4.62754600	3.68785300	-2.31424900
H	5.90957100	4.34275000	-1.26360200
H	5.39145600	2.65257100	-1.08028900
H	4.49515700	6.22717900	-0.52925900
H	3.18457700	5.69099400	-1.60094600
H	2.86433900	5.98532200	0.13060000
C	-4.16700700	-0.48391900	1.30813500
C	-3.10363700	-1.23430000	0.86008000
C	-2.33650000	-0.77811400	-0.23885300
C	-2.71850400	0.41825600	-0.90799500
C	-3.83572800	1.15555600	-0.45524900
C	-4.54246000	0.71528400	0.64641300
H	-4.72391000	-0.80845000	2.19144400
H	-2.83031800	-2.15950200	1.37206100
H	-2.29113100	0.68180200	-1.88565300
H	-4.15735000	2.04596100	-0.99962400

Br	-2.31654600	-2.89523100	-1.82667800
F	-5.58389600	1.82162200	2.44904300
F	-6.85820500	0.73676800	1.10461400
F	-5.96877800	2.59405500	0.47611500
C	-5.73060900	1.46845200	1.15915600

3

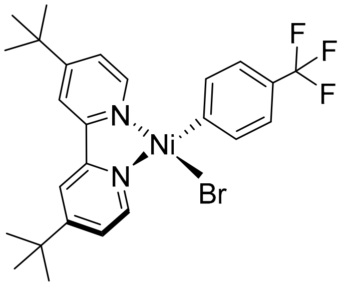

E= -5460.681709
G_{Corr}= 0.416245

C	2.29902900	-2.51101400	0.01548600
C	2.28588700	-0.21064200	0.00321700
C	3.68865500	-2.54786500	-0.00063600
H	1.70549600	-3.42785800	0.02698300
C	3.67392500	-0.17024500	-0.01371600
H	4.17714000	-3.52135900	-0.00089100
H	4.18247800	0.79338200	-0.02502100
C	-0.73408800	1.78767700	0.03324200
C	1.42626600	0.99416900	0.00933600
C	-0.29846900	3.10670800	0.03090700
H	-1.80064000	1.56728000	0.04449300
C	1.92832000	2.28804000	0.00436700
H	-1.05244900	3.89278500	0.04114800
H	3.00694800	2.43858400	-0.00760300
Ni	-0.38499000	-1.14573700	0.02614100
N	1.60528700	-1.37300600	0.01678000
N	0.09735800	0.74070000	0.02292200
C	1.06731600	3.39266400	0.01621800
C	4.41902000	-1.35769800	-0.01692300
C	5.94732800	-1.30668900	-0.04136500
C	6.39777500	-0.58052400	-1.32169300
C	6.44180900	-0.53450200	1.19493100
C	6.56470000	-2.70903500	-0.02608500
H	6.04084900	-1.10633100	-2.22118500
H	6.02675600	0.45477700	-1.36056600
H	7.49763000	-0.54285200	-1.36278400
H	6.11111300	-1.02279900	2.12507200
H	7.54258100	-0.50293600	1.19948800
H	6.07932100	0.50445300	1.20566900
H	7.66195800	-2.62617600	-0.04235500
H	6.28716900	-3.26975600	0.87988300
H	6.26360400	-3.30006300	-0.90478800
C	1.63524100	4.81133900	0.01362600
C	2.51745100	4.99476100	1.26155000
C	2.48424000	5.00316000	-1.25580600
C	0.52611300	5.86792900	0.03204500
H	1.93573800	4.83980100	2.18378500
H	3.36834000	4.29688200	1.27184900
H	2.92471700	6.01764800	1.28332300
H	1.87822000	4.85507900	-2.16339400
H	2.89173500	6.02588100	-1.28110200
H	3.33388300	4.30467500	-1.29332500
H	0.97643800	6.87203100	0.03032100
H	-0.12533600	5.79427200	-0.85239800
H	-0.10231300	5.78692000	0.93238300
Br	-0.90084900	-3.41859000	0.03300600

C	-2.23146100	-0.78767600	0.02222500
C	-2.95387900	-0.61934600	1.21406500
C	-2.93656600	-0.61393400	-1.18269000
C	-4.30575200	-0.27369400	1.21477600
H	-2.45510100	-0.75347900	2.17898900
C	-4.28506300	-0.26916300	-1.20077700
H	-2.42221900	-0.74371800	-2.14000600
C	-4.97589200	-0.09408000	0.00377700
H	-4.83691600	-0.14373800	2.16018700
H	-4.80403700	-0.13475200	-2.15411000
C	-6.42561900	0.28339900	-0.03894000
F	-6.62777600	1.44037400	-0.69545100
F	-7.17153200	-0.63615200	-0.67722100
F	-6.96068000	0.43426600	1.17867900

TS_{3-4}

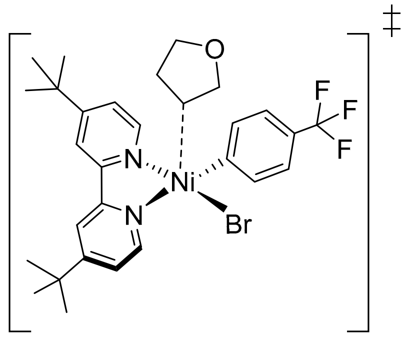

E= -5692.409666
G_{Corr}= 0.515405

C	-2.31653600	-2.37530700	-0.49013700
C	-2.37314800	-0.07635400	-0.39114900
C	-3.70206100	-2.45103500	-0.57840700
H	-1.69496100	-3.27349200	-0.49792200
C	-3.75811300	-0.07526000	-0.49252700
H	-4.16068100	-3.43692500	-0.64208100
H	-4.29571900	0.87209500	-0.49442300
C	0.56417100	1.99423100	0.05651700
C	-1.55976500	1.15054000	-0.22286900
C	0.08266900	3.29082000	0.18798200
H	1.63410900	1.79717600	0.12050900
C	-2.10800100	2.42110700	-0.10652900
H	0.80335700	4.08964000	0.35694200
H	-3.18884400	2.54488900	-0.16893300
Ni	0.33130900	-0.94929400	-0.30802000
N	-1.66008300	-1.21934100	-0.40208400
N	-0.22766900	0.93932900	-0.15049800
C	-1.28822900	3.53824900	0.10116700
C	-4.46519600	-1.28170600	-0.58882200
C	-5.99077000	-1.27022700	-0.69465100
C	-6.57143600	-0.56866900	0.54650200
C	-6.39267000	-0.49769100	-1.96379400
C	-6.56749300	-2.68743500	-0.77487900
H	-6.27869200	-1.09242300	1.47009100
H	-6.23674700	0.47680500	0.62505100
H	-7.67129900	-0.56314000	0.49211100
H	-5.97489300	-0.97308700	-2.86513800
H	-7.48942200	-0.48450800	-2.06239700
H	-6.04721500	0.54683900	-1.93659200
H	-7.66418000	-2.63298200	-0.84867800
H	-6.20152300	-3.23013000	-1.66014200
H	-6.32377700	-3.28077300	0.11997500
C	-1.90469500	4.93153900	0.22498100
C	-2.65574900	5.25652200	-1.07873000
C	-2.88954200	4.93604100	1.40783400
C	-0.84000800	6.00705900	0.46379400

H	-1.97308300	5.24130100	-1.94282600
H	-3.47048500	4.54276100	-1.27344000
H	-3.10042900	6.26182100	-1.01306700
H	-2.37697800	4.68609700	2.35005700
H	-3.33673000	5.93629300	1.51789800
H	-3.71056100	4.21760600	1.26339500
H	-1.32581600	6.99081000	0.54897500
H	-0.28068000	5.83057600	1.39552600
H	-0.11963100	6.06293900	-0.36689800
C	2.17641800	-0.52651400	-0.38425500
C	3.09763900	-0.68589600	0.66177600
C	2.66857100	0.00589900	-1.59203500
C	4.43761300	-0.31740600	0.52337800
H	2.77455900	-1.09310400	1.62382300
C	4.00179000	0.37190400	-1.74889000
H	1.99179200	0.14952200	-2.44094200
C	4.89531800	0.21217700	-0.68338900
H	5.12687300	-0.44645400	1.36095800
H	4.34996100	0.78423100	-2.70021300
C	6.32626700	0.61608500	-0.86882600
F	6.92359800	-0.07107800	-1.85941500
F	6.44693800	1.91496500	-1.19921500
F	7.06779000	0.42856700	0.22966000
C	-0.17096300	-0.98607100	2.40237200
C	0.09437600	-2.39953400	2.82835000
H	-1.17126500	-0.62865900	2.15170000
C	1.10453700	-2.20119900	3.96679600
H	0.53110300	-2.98889200	2.00177900
H	-0.81535300	-2.92645400	3.15726700
H	1.86919400	-2.98857100	4.02866200
H	0.58119800	-2.14867600	4.94217100
O	1.75363000	-0.97332700	3.69419100
C	0.78892500	-0.10300600	3.13710500
H	0.26238600	0.46494900	3.93673300
H	1.30594500	0.63837300	2.50381100
Br	0.90110300	-3.18413700	-0.84988300

4

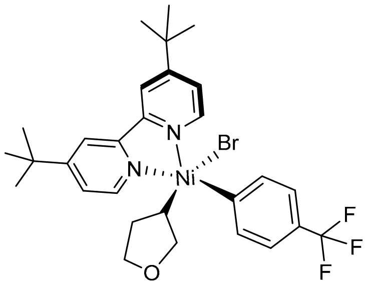

E = -5692.421692

G_{Corr}= 0.517088

C	-1.66056200	-2.64253700	0.03780800
C	-2.35952300	-0.44562100	-0.01468100
C	-2.96472700	-3.11391000	-0.04746500
H	-0.81731400	-3.33586200	0.08713500
C	-3.68849500	-0.84465500	-0.11351500
H	-3.12765400	-4.19070100	-0.05288700
H	-4.47331200	-0.09191000	-0.18113200
C	-0.13383300	2.40718200	0.22607800
C	-1.92338300	0.97597200	0.03922000
C	-0.96299400	3.52300700	0.27054200
H	0.94777000	2.52460300	0.30947000
C	-2.81436200	2.04321400	0.07388800
H	-0.50274600	4.50426100	0.37802900
H	-3.88564200	1.85017300	0.02788900
Ni	0.53456400	-0.62023000	-0.01080100
N	-1.36785500	-1.34570100	0.05943600
N	-0.59341300	1.16700600	0.09284200
C	-2.34714600	3.36003400	0.18025600
C	-4.02385100	-2.20513900	-0.13002200
C	-5.48968000	-2.63078900	-0.23655200
C	-6.26580800	-2.04529100	0.95664400
C	-6.07092200	-2.08409100	-1.55274600
C	-5.64423000	-4.15518300	-0.22475300
H	-5.85559700	-2.40835500	1.91214500
H	-6.23614200	-0.94528800	0.96732100
H	-7.32260400	-2.35014600	0.90174800
H	-5.51666700	-2.47271100	-2.42150500
H	-7.12329800	-2.39249900	-1.65384900
H	-6.04051200	-0.98466300	-1.59137000
H	-6.71100000	-4.41580300	-0.29714800
H	-5.12891500	-4.62698300	-1.07561500
H	-5.25594900	-4.59981400	0.70465600
C	-3.33804200	4.52533600	0.20806600
C	-4.17885100	4.49432200	-1.08067800
C	-4.25816900	4.36622300	1.43173200
C	-2.62499400	5.87849800	0.29896300
H	-3.53944700	4.58484000	-1.97284800
H	-4.76176400	3.56527300	-1.17020400
H	-4.88963900	5.33545100	-1.08390400
H	-3.67623100	4.36422600	2.36679600
H	-4.97078800	5.20473300	1.47556400
H	-4.84216800	3.43436300	1.38981700
H	-3.37196700	6.68663600	0.30650300
H	-2.03022500	5.96832000	1.22101800
H	-1.95870600	6.04928700	-0.56078600
C	2.43547200	-0.30618800	-0.06394700
C	3.35417100	-1.30459700	0.28188200
C	2.95651500	0.88616600	-0.59441000
C	4.73019100	-1.12046800	0.14109500
H	3.01047900	-2.26392500	0.67547100
C	4.32616400	1.09367200	-0.72539900
H	2.28665500	1.67208000	-0.94799900
C	5.22252600	0.08696900	-0.35081900
H	5.41706400	-1.92105900	0.42307600
H	4.69788700	2.03674100	-1.13544400
C	6.69379500	0.32548700	-0.50888800
F	7.03838900	0.53456500	-1.79224200
F	7.10444100	1.41461500	0.16470600
F	7.43667200	-0.69938300	-0.07409000
C	0.64772200	-0.71656400	1.94958600
C	0.84387300	-2.18207600	2.36058000
H	-0.36027300	-0.35914600	2.19943900
C	1.86914500	-2.09584000	3.49751600
H	1.23718500	-2.79766400	1.53337100
H	-0.09535400	-2.65304000	2.68437300
H	2.57107000	-2.94230200	3.52707600
H	1.35001200	-2.04533700	4.47666400
O	2.60014800	-0.91550500	3.26635800
C	1.68246000	0.04161400	2.78988000
H	1.16803400	0.52971100	3.64509800
H	2.24235400	0.82046400	2.25367900
Br	0.49242900	-0.95226500	-2.44287200

5

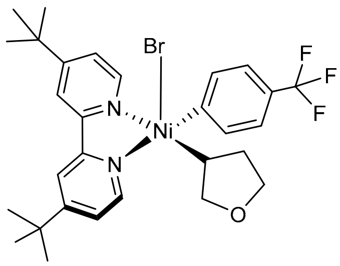

E=-5692.424613
G_{Corr}= 0.517272

C	-2.30956400	-2.36754400	-0.18901900
C	-2.35206700	-0.06876900	-0.24841900
C	-3.67452900	-2.45013100	-0.40956000
H	-1.71992300	-3.27763300	-0.06093800
C	-3.72764300	-0.07449100	-0.47883300
H	-4.13719800	-3.43695900	-0.45574500
H	-4.25341600	0.87098000	-0.58588100
C	0.50973400	2.09600400	0.31258300
C	-1.56625200	1.18600300	-0.10133800
C	0.00673400	3.38572400	0.27066300
H	1.56891400	1.91597800	0.50536600
C	-2.14037800	2.45592200	-0.16276800
H	0.69203500	4.21870800	0.43385500
H	-3.20727400	2.55075900	-0.34989300
Ni	0.37689900	-0.92572900	0.24747400
N	-1.65346800	-1.20805800	-0.11677400
N	-0.25529800	1.01804300	0.12522100
C	-1.35610500	3.59688900	0.02242100
C	-4.42757500	-1.27976400	-0.56737200
C	-5.93473000	-1.35216000	-0.81432800
C	-6.59470500	-2.07203800	0.37536600
C	-6.56169800	0.03821000	-0.95994800
C	-6.18550300	-2.14825900	-2.10747100
H	-6.21907300	-3.09953100	0.49392900
H	-6.41212100	-1.52972200	1.31641300
H	-7.68348000	-2.12936900	0.22040600
H	-6.13767600	0.59339800	-1.81096700
H	-7.64286700	-0.06469700	-1.13704900
H	-6.43113900	0.64498700	-0.05058600
H	-7.26700900	-2.20765600	-2.30630500
H	-5.70629500	-1.66101700	-2.97120200
H	-5.80130700	-3.17707100	-2.03769600
C	-1.92456200	5.01555900	-0.03264800
C	-3.43160300	5.02237200	-0.30916900
C	-1.66555900	5.70508600	1.31888200
C	-1.21018500	5.78840600	-1.15584000
H	-3.67425000	4.55841300	-1.27770400
H	-3.99630300	4.49846100	0.47749300
H	-3.79310800	6.06125800	-0.34012500
H	-0.59109800	5.77681200	1.54522200
H	-2.07233000	6.72829000	1.30057500
H	-2.15335800	5.15872200	2.14139400
H	-1.60747800	6.81371700	-1.21628800
H	-0.12620100	5.85941600	-0.98032600
H	-1.36821200	5.30342400	-2.13202400
C	2.20634200	-0.52108700	-0.02835100
C	2.60209900	-0.19915600	-1.33290200
C	3.13167200	-0.36102500	1.00920800
C	3.88997600	0.26298900	-1.60124200
H	1.90029500	-0.29899500	-2.16678800
C	4.42055100	0.10113300	0.75218100
H	2.83194000	-0.60220600	2.03190700
C	4.80390400	0.41064600	-0.55602300
H	4.17949400	0.50838500	-2.62562800
H	5.13141900	0.21814900	1.57354100
C	6.17305700	0.95963700	-0.83126500
F	6.19801200	2.30437900	-0.78132700
F	7.08291300	0.53312200	0.05497000
F	6.62276600	0.62111900	-2.04783800
C	0.92918100	-2.77158000	-0.00562300
C	2.21329500	-3.44714400	0.45669600
H	0.10464200	-3.21550800	0.57802400
C	2.08318900	-4.79676100	-0.24235100
H	2.28707600	-3.52439500	1.55026000
H	3.10830500	-2.92738700	0.08394500
H	1.46235700	-5.49438700	0.35355300
H	3.05367800	-5.28557700	-0.42694300
O	1.45774800	-4.52650000	-1.48798200
C	0.85673300	-3.23485000	-1.45417800
H	1.41732100	-2.55840100	-2.12662100
H	-0.17507500	-3.30228700	-1.83773200
Br	0.11053900	-1.01231500	2.75993300

6

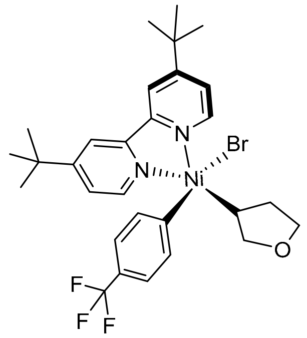

E = -5692.427673
G_{Corr}= 0.516779

C	0.09558800	1.78681100	-1.92397800
C	1.53586900	0.79665300	-0.41972700
C	0.79623600	2.98612300	-1.88376500
H	-0.78967000	1.67498900	-2.55506200
C	2.28550300	1.96496300	-0.32880900
H	0.44152300	3.81440000	-2.49555800
H	3.15893900	1.99469200	0.32210600
C	1.24957000	-2.64178700	0.70308700
C	1.85815900	-0.45330700	0.32024400
C	2.32438600	-2.84378600	1.56199800
H	0.55022200	-3.45372600	0.49260900
C	2.95106300	-0.58044200	1.17013100
H	2.44753900	-3.82625800	2.01553800
H	3.61131600	0.27190100	1.32736400
Ni	-0.56054400	-1.04690600	-1.18321600
N	0.45413600	0.72468500	-1.21045400
N	1.01490500	-1.47836400	0.10335000
C	3.20997700	-1.79471400	1.82056700
C	1.92497600	3.10048300	-1.06670900
C	2.75531800	4.38083500	-0.95489100
C	2.76104600	4.84376100	0.51295300
C	4.19445900	4.08025800	-1.41050700
C	2.18772000	5.50752100	-1.82439900
H	1.73829200	5.04747200	0.86726800
H	3.21092500	4.09314900	1.18017900
H	3.34682600	5.77116000	0.61071800
H	4.21352200	3.72905400	-2.45420300
H	4.80605000	4.99392500	-1.34701300
H	4.67335800	3.31345400	-0.78301000
H	2.81155300	6.40711900	-1.71162600
H	2.18203400	5.23925700	-2.89216500
H	1.16130400	5.77562800	-1.52970100
C	4.41557000	-1.92189100	2.75336200
C	5.69712300	-1.63598000	1.95026000
C	4.27422800	-0.89428000	3.89037100
C	4.51887800	-3.32298000	3.36481700
H	5.81060000	-2.35001400	1.11944000
H	5.70243900	-0.61886400	1.53020600
H	6.57770800	-1.73152400	2.60464400
H	3.35250700	-1.06775800	4.46777900
H	5.12899200	-0.97857100	4.57957100
H	4.25163600	0.13892300	3.51223000
H	5.39539100	-3.36903100	4.02871300
H	3.63218400	-3.57420600	3.96722000
H	4.64495000	-4.09910900	2.59416500

C	-1.83431000	-0.26025700	0.00424300
C	-1.65503900	-0.17771500	1.38789400
C	-2.91072100	0.43164600	-0.56743000
C	-2.53136100	0.55674400	2.18794600
H	-0.82365800	-0.70015100	1.86976300
C	-3.79220500	1.16842000	0.21755100
H	-3.08451400	0.38539900	-1.64708300
C	-3.60428000	1.22885900	1.60259800
H	-2.37447300	0.60356000	3.26734100
H	-4.63131500	1.69278800	-0.24728300
C	-4.57295200	2.02142700	2.42938700
F	-4.61872200	3.31116100	2.05315700
F	-5.82944500	1.55745700	2.31531300
F	-4.27461900	2.01015700	3.73360500
C	-1.64112500	-2.68152800	-1.05142800
C	-2.22739200	-3.21272000	0.25324100
H	-0.86730200	-3.35190300	-1.45032300
C	-3.69626600	-2.78112600	0.20831100
H	-1.71615500	-2.85424900	1.15798800
H	-2.15433600	-4.31398000	0.25546400
H	-3.88364000	-1.89617600	0.84087800
H	-4.36863200	-3.58580500	0.55522900
O	-3.99315400	-2.45686500	-1.13718500
C	-2.86271800	-2.64146200	-1.96106200
H	-2.94867600	-3.59665300	-2.52180900
H	-2.83249700	-1.83572500	-2.71346700
Br	0.32124300	-1.64203600	-3.41248700

7

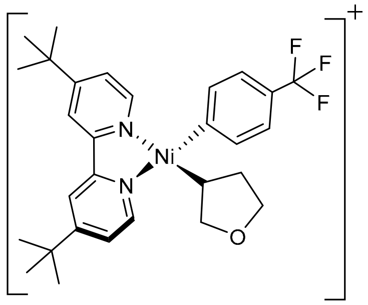

E= -3118.159085
G_{Corr}= 0.520015

C	2.23753500	-2.50894200	-0.38915400
C	2.34669900	-0.21918000	-0.14682200
C	3.62013700	-2.63167600	-0.33686300
H	1.61694800	-3.39832300	-0.51711100
C	3.73219600	-0.26880900	-0.08245300
H	4.05159000	-3.62759400	-0.42480600
H	4.29564200	0.65541500	0.03751600
C	-0.52134400	2.01264500	-0.16839400
C	1.57598700	1.05095600	-0.08557100
C	0.00727300	3.29099800	-0.04130200
H	-1.59929300	1.86485100	-0.25680300
C	2.16940100	2.29804100	0.05124400
H	-0.68176200	4.13418400	-0.03530800
H	3.25250900	2.37147100	0.13871000
Ni	-0.42103100	-1.00737800	-0.24457600
N	1.60762000	-1.33922800	-0.28778900
N	0.23844100	0.91739300	-0.18682300
C	1.38786400	3.46230600	0.08047600
C	4.41070600	-1.49295700	-0.17026800
C	5.93622200	-1.53126600	-0.08409700
C	6.52025900	-0.69398700	-1.23633900
C	6.36895900	-0.93242700	1.26651200
C	6.47715400	-2.96090200	-0.18756700
H	6.20299000	-1.08992100	-2.21374300
H	6.21341100	0.36117700	-1.17722500
H	7.62023000	-0.72399500	-1.19700900
H	5.94672200	-1.50556100	2.10678900
H	7.46636400	-0.96112500	1.35152300
H	6.05342000	0.11640100	1.37359600
H	7.57490200	-2.94247800	-0.11422000
H	6.10039600	-3.60180100	0.62444700
H	6.21652800	-3.42998000	-1.14886200
C	2.05656200	4.82684200	0.24650600
C	2.80001700	4.84600800	1.59454500
C	3.05878300	5.03701800	-0.90276000
C	1.03557300	5.96892700	0.22693200
H	2.10431300	4.68224500	2.43241100
H	3.58346800	4.07465200	1.64326400
H	3.28403600	5.82439500	1.73926400
H	2.55382100	4.99867500	-1.88067600
H	3.53516500	6.02469500	-0.80354800
H	3.85832200	4.28091700	-0.89892200
H	1.55900100	6.92919200	0.34824100
H	0.48325700	6.00871700	-0.72470500
H	0.30657500	5.88227700	1.04731900
C	-2.25663700	-0.61866000	-0.16227400
C	-2.87271100	-0.38210300	1.06624400
C	-2.95321600	-0.37040000	-1.34906800
C	-4.18113500	0.10106100	1.11462200
H	-2.34172100	-0.57236600	2.00319000
C	-4.25772500	0.11228700	-1.30603600
H	-2.48392800	-0.55555800	-2.31923000
C	-4.87246200	0.34678800	-0.07149800
H	-4.65783500	0.28615100	2.07890500
H	-4.79854200	0.30511800	-2.23603100
C	-6.28339100	0.86295000	-0.05639500
F	-6.39437300	2.03259600	-0.70695200
F	-7.13150900	0.01615300	-0.66220200
F	-6.74903600	1.06072800	1.18094600
C	-1.01904400	-2.85507700	-0.14729900
C	-2.37231400	-3.48868300	-0.45170300
H	-0.28905400	-3.26653800	-0.86707000
C	-2.18861500	-4.84746000	0.21459500
H	-2.59039900	-3.55771100	-1.52655900
H	-3.19942000	-2.95448200	0.03702900
H	-1.63222100	-5.54214200	-0.44487100
H	-3.14094300	-5.32830700	0.48659000
O	-1.45226700	-4.58866600	1.39849500
C	-0.74778100	-3.36221100	1.26770400
H	-1.12082700	-2.64905000	2.02810800
H	0.32545800	-3.52268400	1.46499700

8

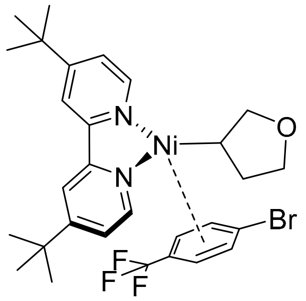

E = -5692.39712
G_{Corr}= 0.514089

C	1.97811500	-2.31678200	-0.50260200
C	1.52565000	-0.08774400	-0.84764600
C	3.32623500	-2.05850200	-0.27496400
H	1.59017200	-3.33787800	-0.45555400
C	2.85887300	0.24677700	-0.64449700
H	3.98354000	-2.89614800	-0.04474800

H	3.16854500	1.28935100	-0.71354500
C	-1.75648300	1.17923900	-1.71125100
C	0.45654600	0.90130800	-1.14059400
C	-1.63215100	2.55835100	-1.71310900
H	-2.71207800	0.70240300	-1.94165100
C	0.65383900	2.28183300	-1.11384400
H	-2.50945900	3.16292300	-1.94885500
H	1.63579000	2.67004600	-0.85214500
Ni	-0.90090200	-1.59442100	-1.13378900
N	1.09501200	-1.36038900	-0.77729400
N	-0.74174500	0.35797400	-1.41596400
C	-0.40330000	3.15076500	-1.38789800
C	3.79921700	-0.74516000	-0.33633300
C	5.25213000	-0.35837800	-0.05473500
C	5.27684900	0.55943700	1.18165200
C	6.12678900	-1.58493000	0.22450900
C	5.82465800	0.39070600	-1.27037600
H	4.71456500	1.48998200	1.01261200
H	4.84208800	0.05360400	2.05766400
H	6.31527300	0.83439400	1.42519300
H	6.14308000	-2.27983000	-0.62950500
H	7.16255300	-1.26289800	0.41128000
H	5.78701100	-2.13796000	1.11376100
H	6.87128000	0.67431800	-1.07782200
H	5.80389600	-0.24307100	-2.17100900
H	5.26546700	1.31292900	-1.48890300
C	-0.27293200	4.67246000	-1.30982500
C	-1.23020400	5.18350400	-0.21740400
C	1.15275000	5.10979300	-0.95754000
C	-0.65911200	5.28381900	-2.66748600
H	-2.27909500	4.93985200	-0.44472800
H	-0.98049200	4.74575000	0.76208900
H	-1.15227100	6.27872500	-0.12969500
H	1.88318600	4.77188400	-1.70898200
H	1.20090800	6.20857400	-0.91769700
H	1.46796300	4.72889700	0.02638000
H	-0.57375200	6.38103200	-2.62446100
H	0.00587900	4.92142900	-3.46730700
H	-1.69496600	5.04066900	-2.94812600
C	-1.59207400	-3.44661000	-1.07295600
C	-1.37705300	-4.24713700	0.22342500
C	-0.99147300	-4.42823900	-2.06417100
H	-2.69036400	-3.45516100	-1.25689600
C	-1.63444400	-5.69028100	-0.22301300
H	-2.03717700	-3.94839500	1.05317400
H	-0.33664900	-4.15097700	0.58535200
H	0.10954300	-4.31165600	-2.15171200
H	-1.40843000	-4.33289400	-3.08244400
H	-1.05754800	-6.43570800	0.35443800
H	-2.70773200	-5.94674000	-0.11118200
O	-1.26038500	-5.75670300	-1.58527100
C	-0.90580900	1.61203600	1.94229600
C	-2.23633800	1.59454400	1.54155900
C	-2.86902700	0.36651900	1.34801200
C	-2.19378200	-0.83543000	1.53612100
C	-0.86034200	-0.80860600	1.94271600
C	-0.22097300	0.41121900	2.14850000
H	-0.40309500	2.56933000	2.09250100
H	-2.76823800	2.53250800	1.37583300
H	-2.69725300	-1.78973700	1.38164900
H	-0.32749100	-1.74896500	2.09189500
C	1.19983700	0.45615200	2.63807500
F	1.88185700	1.48250300	2.10899600
F	1.87662300	-0.66064600	2.34865100
F	1.26243200	0.60864800	3.97058200
Br	-4.67266700	0.33443400	0.77286800

9

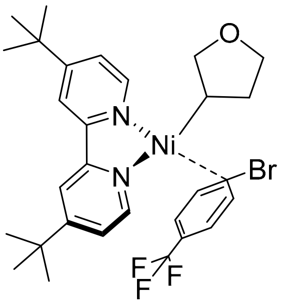

E=-5692.383727

G_{Corr}= 0.514794

C	-0.37964000	2.31327500	0.55587300
C	1.49883600	1.04347800	0.13965700
C	0.36172800	3.48814800	0.60200800
H	-1.46421200	2.33062600	0.69456900
C	2.30504300	2.17738800	0.17330800
H	-0.16213400	4.42499700	0.78737000
H	3.37814600	2.07940600	0.01022400
C	1.45497800	-2.55213700	-0.35152700
C	2.01167800	-0.33149200	-0.10971800
C	2.78347100	-2.91994500	-0.53129100
H	0.66140000	-3.30292600	-0.36922200
C	3.36132000	-0.62524700	-0.27882900
H	3.01212600	-3.97281300	-0.69126600
H	4.09488700	0.17962600	-0.23629100
Ni	-0.85971700	-0.65445800	0.27385000
N	0.17250100	1.12304700	0.33245900
N	1.07123900	-1.29314300	-0.15208400
C	3.78267400	-1.94316300	-0.49849500
C	1.74605900	3.44063200	0.40916600
C	2.64230500	4.68083600	0.44014700
C	3.70372700	4.50307300	1.54040200
C	3.33278000	4.82832200	-0.92757400
C	1.84300200	5.95589300	0.72874000
H	3.23276700	4.37327800	2.52751200
H	4.35034400	3.63257100	1.35262000
H	4.34958000	5.39399200	1.58532800
H	2.59164100	4.94092000	-1.73451700
H	3.97608300	5.72226500	-0.92863100
H	3.96696700	3.96067500	-1.16458000
H	2.52438900	6.82000400	0.74535300
H	1.08279400	6.14794400	-0.04416100
H	1.33867900	5.91143100	1.70646300
C	5.27012900	-2.25227400	-0.68099100
C	5.79761900	-1.46334700	-1.89267600
C	6.02573700	-1.81991700	0.58836200
C	5.51851400	-3.74546200	-0.91844000
H	5.25803500	-1.74199500	-2.81154800
H	5.69540200	-0.37663500	-1.75230400
H	6.86628600	-1.68101700	-2.04563700
H	5.65516300	-2.36165600	1.47280100
H	7.09940700	-2.03945000	0.47901300
H	5.92300800	-0.74117400	0.78064000
H	6.59733100	-3.92212900	-1.04561400
H	5.17970300	-4.35829600	-0.06880100
H	5.01335600	-4.10577800	-1.82802200
C	-2.31526000	-1.56440000	-0.74844200
C	-1.63223000	-0.66301300	-1.65104500
C	-3.59820100	-1.15271200	-0.22871600
C	-2.18711200	0.62031200	-1.89373700
H	-0.85059300	-1.02944900	-2.31985300
C	-4.06661900	0.11300200	-0.43917000
H	-4.17947200	-1.85618000	0.37102600
C	-3.34194700	1.02799100	-1.26776000
H	-1.66597300	1.28933900	-2.58130900
H	-5.01128700	0.42110600	0.01512100

C	-3.87100500	2.41291900	-1.43580100
F	-5.12083800	2.43084600	-1.93419800
F	-3.94186700	3.06908500	-0.25801000
F	-3.11969900	3.16642300	-2.24685300
C	-1.05403300	-1.16642800	2.20453000
C	-1.99860800	-0.23257800	2.97131500
H	-0.05378600	-1.08656900	2.68107000
C	-2.25583200	-1.00462500	4.27078900
H	-2.95143600	-0.09756700	2.42429200
H	-1.58226900	0.77022400	3.15690700
H	-3.26038600	-0.82028700	4.69376200
H	-1.51548200	-0.72140700	5.04577100
O	-2.11900700	-2.37665700	3.95363500
C	-1.64345800	-2.50155200	2.60394100
H	-0.91916100	-3.33379000	2.56338900
H	-2.49993000	-2.78358800	1.95841700
Br	-2.12416300	-3.47245700	-1.03662900

TS$_{9-4}$

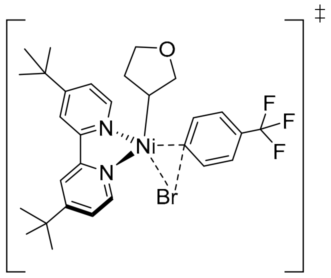

E= -5692.363606
G_{Corr}= 0.513587

C	0.74697500	2.77349100	-0.18961600
C	2.13414000	0.93590800	-0.04228800
C	1.82105700	3.65452800	-0.13682000
H	-0.27753500	3.14637800	-0.26784500
C	3.25532600	1.75671900	0.01447400
H	1.61791200	4.72377800	-0.17740900
H	4.24671700	1.31238400	0.09720000
C	0.99216200	-2.51484900	-0.00083200
C	2.20171300	-0.55368700	0.01958000
C	2.13979500	-3.28643700	0.14002100
H	0.00838000	-2.98537300	-0.07154400
C	3.39244300	-1.25988300	0.16072500
H	2.03586000	-4.36980600	0.18161100
H	4.33619000	-0.71914200	0.22512000
Ni	-0.64229300	0.05204300	-0.13598700
N	0.89662700	1.45142200	-0.14849100
N	1.01985000	-1.18688100	-0.06073500
C	3.38672400	-2.65988800	0.22274000
C	3.12115100	3.15154400	-0.03484000
C	4.36508100	4.04033100	0.02434900
C	5.10691400	3.76910900	1.34542900
C	5.27921400	3.69389300	-1.16458900
C	4.00849300	5.52883500	-0.04486400
H	4.46591300	3.99311600	2.21256600
H	5.43683000	2.72224400	1.42499200
H	6.00227600	4.40707000	1.40947500
H	4.76283700	3.86147100	-2.12286400
H	6.17677500	4.33167900	-1.14492900
H	5.61392100	2.64595000	-1.13470700
H	4.93005700	6.12876600	-0.00062100
H	3.49082100	5.78373900	-0.98259700
H	3.37036300	5.83764200	0.79751100
C	4.70381300	-3.42428100	0.37278400
C	5.60796600	-3.09516300	-0.82867100
C	5.39130900	-2.98087700	1.67645900
C	4.48221200	-4.93957100	0.42264200
H	5.12838100	-3.38724600	-1.77618500
H	5.84634400	-2.02211100	-0.88271100
H	6.55861800	-3.64467700	-0.74414900
H	4.75502300	-3.19116100	2.55050800
H	6.33871800	-3.52740200	1.80512600
H	5.62440800	-1.90532100	1.67381900
H	5.45198100	-5.44842500	0.53144500
H	3.85458700	-5.23472300	1.27771900
H	4.01119700	-5.31618000	-0.49852500
C	-2.24366100	-0.55634100	-1.13499600
C	-2.66594200	0.80038800	-1.29304300
C	-3.21493300	-1.52257800	-0.73787700
C	-3.94515900	1.18652700	-0.89537300
H	-2.01068800	1.53695100	-1.76642900
C	-4.47469600	-1.12024600	-0.35882200
H	-2.94466100	-2.58007200	-0.70267000
C	-4.85250200	0.24187400	-0.42179600
H	-4.23695600	2.23519800	-0.98266800
H	-5.18981600	-1.86616300	-0.00263400
C	-6.18944100	0.65059500	0.09691700
F	-7.17519700	-0.16722400	-0.31135500
F	-6.23725200	0.63105400	1.44755900
F	-6.53465900	1.89122200	-0.27160300
C	-1.16611700	-0.10450400	1.80331300
C	-2.36727500	0.69590200	2.31089700
H	-0.26923100	0.24789600	2.35640400
C	-2.64076200	0.05048700	3.66940100
H	-3.24168800	0.55298700	1.65605000
H	-2.18221800	1.77924500	2.38551800
H	-3.69889500	0.11541600	3.97940200
H	-2.03212400	0.53251200	4.46208500
O	-2.28032100	-1.31065200	3.53288800
C	-1.51418200	-1.48233000	2.33043400
H	-0.62353900	-2.09404200	2.55988200
H	-2.13459900	-2.05900600	1.61490900
Br	-0.97307600	-1.14685100	-2.70484500

TS$_{7-10`}$

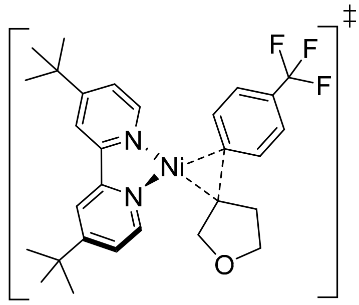

E=-3118.154505
G_{Corr}= 0.520596

C	2.23025700	-2.53460500	-0.18624800
C	2.36358900	-0.23495100	-0.06202600
C	3.61187300	-2.66067600	-0.27915500
H	1.58758100	-3.41986200	-0.20092300
C	3.74823900	-0.28382200	-0.15509400
H	4.03667600	-3.65980800	-0.36436900
H	4.32099100	0.64303700	-0.15128500
C	-0.51927400	1.97675900	0.11077800
C	1.59150700	1.03796900	0.04480800
C	0.00147400	3.26192500	0.20502500
H	-1.59981000	1.80653800	0.09784900
C	2.18044100	2.29172800	0.14542500
H	-0.69246000	4.09914700	0.26267800
H	3.26664500	2.37752100	0.16596300

Ni	-0.42436100	-1.02157600	-0.02609900
N	1.62059400	-1.35722700	-0.07820100
N	0.25350000	0.89651900	0.03384800
C	1.38650000	3.44636000	0.22761900
C	4.41185300	-1.51540200	-0.26618500
C	5.93719900	-1.55440800	-0.37104100
C	6.36853000	-0.76398200	-1.61957100
C	6.53780200	-0.90583100	0.88925000
C	6.46543400	-2.98785300	-0.48804000
H	5.93457300	-1.19987000	-2.53315900
H	6.06365700	0.29198100	-1.56561800
H	7.46502900	-0.79007100	-1.71711000
H	6.22744600	-1.44535200	1.79781400
H	7.63724900	-0.93399900	0.83503900
H	6.23624700	0.14712300	0.99614600
H	7.56323400	-2.96808200	-0.56140800
H	6.20221400	-3.59552700	0.39150200
H	6.08144500	-3.49468900	-1.38685100
C	2.04826000	4.82110900	0.33388400
C	2.93311400	4.85040800	1.59297000
C	2.91518200	5.05073000	-0.91749100
C	1.01398300	5.94746500	0.43072800
H	2.33607200	4.66866000	2.50040200
H	3.73356300	4.09630900	1.55264000
H	3.40989400	5.83811500	1.69075200
H	2.30456700	5.01575800	-1.83338000
H	3.39202800	6.04181900	-0.86351100
H	3.71492200	4.30041500	-1.01008800
H	1.53302400	6.91457600	0.50938200
H	0.36740200	5.98902900	-0.45943500
H	0.37306500	5.84078500	1.31956400
C	-2.28456100	-0.97259500	-0.06049100
C	-2.96737600	-0.58024000	1.09966800
C	-2.84657600	-0.67786700	-1.31646200
C	-4.18243300	0.09552500	1.00963600
H	-2.55229300	-0.79735100	2.08700200
C	-4.05424800	-0.00033400	-1.40533100
H	-2.33747700	-0.99178500	-2.23194100
C	-4.72961600	0.38511000	-0.24020800
H	-4.69998400	0.39915800	1.92131600
H	-4.47759000	0.22658800	-2.38698300
C	-6.04042900	1.10416800	-0.36779500
F	-5.92362600	2.23774900	-1.07954500
F	-6.96195700	0.35992100	-1.00187800
F	-6.56211000	1.44136200	0.81626800
C	-1.41370800	-2.79118400	0.04099200
C	-2.69642300	-3.56126800	-0.29332100
H	-0.66161500	-3.11814300	-0.70163000
C	-2.36621000	-4.91128400	0.32629500
H	-2.89699600	-3.61256100	-1.37190000
H	-3.57661100	-3.12986500	0.20696300
H	-1.71963500	-5.50897000	-0.34596400
H	-3.26040200	-5.50861300	0.55928600
O	-1.69293600	-4.60969100	1.53428100
C	-1.06886500	-3.33979500	1.42817600
H	-1.45113900	-2.69014200	2.23541300
H	0.02154900	-3.43551800	1.56846400

TS4-10

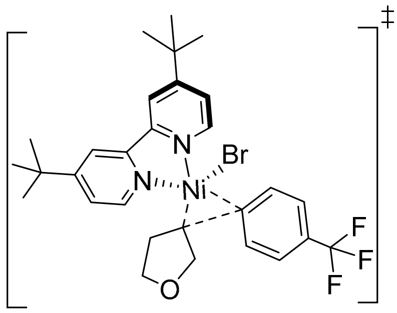

E=-5692.409939

G_{Corr}= 0.515453

C	2.10062300	-2.46920000	0.53508200
C	2.42890300	-0.25249400	0.01670400
C	3.42409400	-2.78698000	0.24780800
H	1.40628300	-3.23268300	0.89473500
C	3.76189100	-0.49357700	-0.30177300
H	3.75284100	-3.81717500	0.37876000
H	4.39689000	0.32946800	-0.62890800
C	-0.07414500	2.33454300	0.48225100
C	1.81823200	1.10682700	0.03187700
C	0.60973300	3.53794000	0.35246700
H	-1.13470800	2.32914700	0.74238500
C	2.56306200	2.27005200	-0.13134500
H	0.06207600	4.46468500	0.51854000
H	3.62747000	2.19879300	-0.35337800
Ni	-0.44721800	-0.70586100	0.47942200
N	1.60909200	-1.24179400	0.40256200
N	0.49954700	1.14690700	0.30012400
C	1.96467400	3.52897800	0.01357400
C	4.29146500	-1.78771900	-0.20247300
C	5.75420100	-2.04673200	-0.56980200
C	5.96884200	-1.66443000	-2.04524700
C	6.65601400	-1.18042900	0.32736100
C	6.13851200	-3.51796100	-0.38099800
H	5.32236100	-2.26240700	-2.70679400
H	5.75652400	-0.60051800	-2.23034500
H	7.01555600	-1.85100200	-2.33246600
H	6.50354100	-1.42085400	1.39131300
H	7.71431000	-1.36469400	0.08461300
H	6.46458100	-0.10564400	0.18879500
H	7.19425700	-3.65971000	-0.65767100
H	6.02306700	-3.84128200	0.66511800
H	5.53612900	-4.18463000	-1.01741100
C	2.79475900	4.79922900	-0.17869200
C	3.94289800	4.80469000	0.84627800
C	3.37242900	4.80085900	-1.60534500
C	1.95402300	6.06543300	0.01468700
H	3.55378900	4.78111700	1.87632800
H	4.61623600	3.94427700	0.71500900
H	4.54413000	5.71999000	0.72976800
H	2.56837600	4.78001600	-2.35776500
H	3.96723900	5.71336600	-1.76753200
H	4.03067600	3.93714500	-1.78386900
H	2.58616300	6.95261000	-0.14178700
H	1.12184200	6.11889200	-0.70431700
H	1.53707500	6.13032300	1.03156400
C	-2.23184700	-0.43917900	-0.18375900
C	-3.27092000	-1.23854300	0.32560300
C	-2.59915700	0.78837100	-0.76984300
C	-4.59536200	-0.81682800	0.30240300
H	-3.03904800	-2.20350400	0.78125100
C	-3.91777200	1.22641800	-0.78798900
H	-1.83965400	1.40947200	-1.24973900
C	-4.92670600	0.42547300	-0.24433200
H	-5.37380500	-1.45802400	0.72123900
H	-4.16131100	2.19282500	-1.23653100

C	-6.35010800	0.88605000	-0.31117000
F	-7.15344100	0.20515400	0.51630900
F	-6.47808000	2.18586500	0.00266200
F	-6.87295400	0.75092100	-1.54514000
C	-0.88457400	-1.48905400	-1.36576000
C	-0.88596100	-2.98292300	-0.99441600
H	0.10208700	-1.10903100	-1.66866700
C	-1.99883200	-3.59296100	-1.86968800
H	-1.08301700	-3.14921300	0.07836700
H	0.08769600	-3.44386300	-1.21081100
H	-2.70381500	-4.21799000	-1.30262400
H	-1.55611900	-4.21583500	-2.67090800
O	-2.71136700	-2.51361700	-2.43056300
C	-1.78882100	-1.47474400	-2.60459300
H	-1.14393800	-1.67635800	-3.48699700
H	-2.32957300	-0.53933000	-2.79342500
Br	-0.77869400	-1.44100700	2.80423900

$TS_{5\text{-}10}$

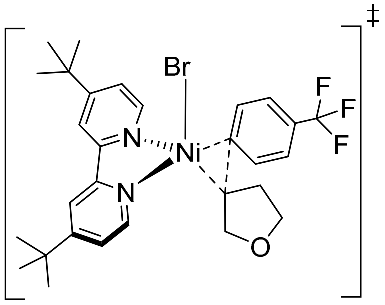

E = -5692.418456

G_{Corr}= 0.516644

C	-1.39921200	-2.75947300	-0.38817500
C	-1.93872800	-0.55735100	0.01074900
C	-2.59197600	-2.98285100	-1.05752800
H	-0.67641200	-3.56881700	-0.25982600
C	-3.15453000	-0.70509000	-0.65682800
H	-2.79243600	-3.97987200	-1.45243600
H	-3.82611600	0.14720900	-0.73124000
C	0.08678400	1.73914200	1.95730200
C	-1.51818400	0.71659700	0.65837800
C	-0.59332000	2.94633200	1.93294400
H	1.02779900	1.62459100	2.50162900
C	-2.25992600	1.89683700	0.59112300
H	-0.16863800	3.79995500	2.46357600
H	-3.19350400	1.90966900	0.03334400
Ni	0.73148300	-1.07884700	1.09139700
N	-1.06905300	-1.57265600	0.12614900
N	-0.36356600	0.65282600	1.33047200
C	-1.80227200	3.05207300	1.22973700
C	-3.50941100	-1.93512600	-1.21604300
C	-4.82463500	-2.16142000	-1.96300900
C	-5.61514600	-3.27179200	-1.24831700
C	-5.68539800	-0.89471200	-2.01037000
C	-4.50364700	-2.59745900	-3.40389400
H	-5.06390400	-4.22397200	-1.23367000
H	-5.84037400	-2.98996000	-0.20769800
H	-6.56993400	-3.44590300	-1.76871300
H	-5.17537700	-0.07000700	-2.53181600
H	-6.61708500	-1.10586400	-2.55658300
H	-5.96325800	-0.54856700	-1.00277300
H	-5.43854000	-2.76122600	-3.96242500
H	-3.92259300	-1.82434100	-3.93094900
H	-3.92917200	-3.53568100	-3.43102600
C	-2.55681700	4.38213800	1.18146000
C	-3.84538900	4.28381900	0.35805900
C	-2.91738800	4.80020000	2.61805800
C	-1.64296100	5.44452200	0.54481000
H	-3.64432100	4.00657000	-0.68839300
H	-4.54942900	3.55072700	0.78138700
H	-4.35053900	5.26148200	0.35036300
H	-2.02225600	4.93742500	3.24316700
H	-3.46558600	5.75529300	2.60433500
H	-3.55909900	4.04585000	3.10003500
H	-2.16987400	6.41025300	0.49358800
H	-0.72164800	5.59681600	1.12694400
H	-1.35695400	5.15744800	-0.47939100
C	1.81699200	-0.39013800	-0.28860500
C	1.31256400	-0.16292200	-1.57781500
C	2.90541200	0.38226900	0.14716200
C	1.86433300	0.81432900	-2.40050300
H	0.47255100	-0.75440400	-1.94940300
C	3.45807900	1.36471300	-0.66829300
H	3.31728000	0.22509200	1.14787500
C	2.93811600	1.58411000	-1.94731800
H	1.45058500	0.97853800	-3.39717600
H	4.29555800	1.96497900	-0.30348100
C	3.55955700	2.63966600	-2.81268700
F	4.83577000	2.35161600	-3.12292000
F	2.91074000	2.80901700	-3.97043800
F	3.58776400	3.83722100	-2.20382800
C	1.98799400	-2.44028300	0.29424300
C	3.38120400	-2.67804700	0.85636200
H	1.28714600	-3.10981600	0.83592200
C	3.71959000	-4.04858100	0.25767400
H	3.40262600	-2.66626800	1.95309600
H	4.08452700	-1.91550400	0.48593300
H	3.48784500	-4.86410200	0.96524700
H	4.78588300	-4.13453200	-0.01461700
O	2.90735600	-4.20511500	-0.90081000
C	2.15002300	-3.02622800	-1.10369600
H	2.69400300	-2.33615700	-1.77543200
H	1.19982100	-3.29370900	-1.58964100
Br	1.28173300	-1.35851700	3.48388700

$TS_{6\text{-}10}$

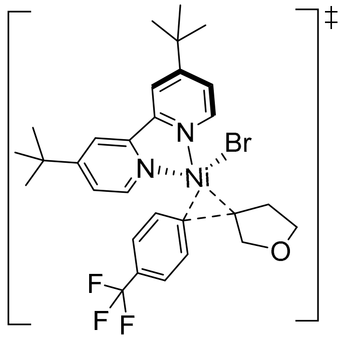

E=-5692.420641

G_{Corr}= 0.516182

C	0.08767500	1.71603900	1.96317400
C	-1.54794900	0.72909600	0.67591900
C	-0.52291900	2.96231900	1.87352100
H	1.01096900	1.57310900	2.53125800
C	-2.21258400	1.94282200	0.53780600
H	-0.05558900	3.80643600	2.37902400
H	-3.12862600	1.99153000	-0.05040200
C	-1.56678900	-2.77591200	-0.27269100
C	-2.02524200	-0.54657000	0.07297100
C	-2.76841000	-2.97340200	-0.94399000
H	-0.87473900	-3.60871800	-0.12668000
C	-3.24335900	-0.66852900	-0.58569200

H	-2.99576300	-3.97149100	-1.31578400
H	-3.89348500	0.20131300	-0.67651300
Ni	0.61862600	-1.15034800	1.18623200
N	-0.40865600	0.63084100	1.37819400
N	-1.19381900	-1.59517300	0.21591200
C	-3.64422600	-1.89981600	-1.12292100
C	-1.70432600	3.10257900	1.13969700
C	-2.43685000	4.43512100	0.96899800
C	-2.48804800	4.78121400	-0.53000600
C	-3.86750500	4.29322900	1.51846000
C	-1.73373100	5.57418700	1.71449100
H	-1.47369500	4.87155400	-0.94955500
H	-3.03096000	4.01916200	-1.10949300
H	-3.00430300	5.74305700	-0.67568200
H	-3.85605900	4.02782100	2.58733600
H	-4.40669700	5.24735400	1.41008200
H	-4.44143400	3.52323300	0.98112900
H	-2.29435400	6.50974800	1.56736800
H	-1.68165400	5.38455200	2.79779700
H	-0.71062600	5.73865200	1.34252800
C	-4.98243000	-2.01716100	-1.85457800
C	-6.11525600	-1.65041600	-0.87928300
C	-4.98525100	-1.04066800	-3.04448700
C	-5.22358400	-3.43589300	-2.38077800
H	-6.12117700	-2.32444600	-0.00830400
H	-6.02379800	-0.61709400	-0.51190500
H	-7.08885000	-1.73969600	-1.38615000
H	-4.16857000	-1.27034500	-3.74688300
H	-5.93773300	-1.12057200	-3.59137200
H	-4.87403900	0.00512200	-2.72036300
H	-6.19097700	-3.47299200	-2.90414000
H	-4.44645000	-3.74565300	-3.09663500
H	-5.25842300	-4.17572200	-1.56615500
C	1.79439900	-0.59707500	-0.19221500
C	1.27982200	-0.33118200	-1.47048500
C	2.94913900	0.09284700	0.20820600
C	1.88836000	0.59000700	-2.31758200
H	0.37972900	-0.84370200	-1.81827200
C	3.56647100	1.01247500	-0.63224900
H	3.36789700	-0.08167300	1.20215700
C	3.03917400	1.26298800	-1.90266200
H	1.46043700	0.78464100	-3.30280500
H	4.46277000	1.53888000	-0.29445900
C	3.70243400	2.28128000	-2.78076400
F	3.53648000	3.53305200	-2.31631400
F	5.02909300	2.08996500	-2.86379500
F	3.22920500	2.27221500	-4.03264800
C	1.77050400	-2.64603400	0.43127800
C	1.79237500	-3.29071900	-0.95105800
H	1.06054300	-3.17721800	1.09473000
C	3.23083700	-3.06412800	-1.41729600
H	1.04843500	-2.89768300	-1.65614400
H	1.59584800	-4.37026900	-0.83781400
H	3.31566700	-2.15657800	-2.04122100
H	3.61202600	-3.91373800	-2.00896100
O	4.01438100	-2.90543500	-0.24784900
C	3.19460300	-2.88836200	0.90266100
H	3.24379100	-3.86799500	1.42105300
H	3.56234600	-2.13419100	1.61269200
Br	1.43146500	-1.25805600	3.53206300

10

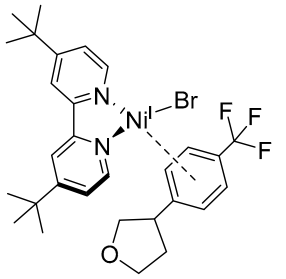

E=-5692.48759

G_{Corr}= 0.517819

C	1.87785800	-1.90144800	-1.28336000
C	1.55577200	0.32160900	-0.77338300
C	3.25324500	-1.77863200	-1.16815600
H	1.40657700	-2.85207700	-1.54788000
C	2.93066100	0.52223200	-0.63738500
H	3.87634000	-2.65794300	-1.33854000
H	3.30308100	1.51089200	-0.37838900
C	-1.69185400	1.93587200	-0.73852200
C	0.54300900	1.40178400	-0.61516300
C	-1.43698900	3.28205600	-0.49855400
H	-2.71214000	1.58907100	-0.90503700
C	0.87210300	2.72670100	-0.35389200
H	-2.27866600	3.97319600	-0.48276800
H	1.91737900	3.00190000	-0.21497000
Ni	-0.98533100	-1.02812500	-0.90412900
N	1.04660300	-0.87989700	-1.07345500
N	-0.73640500	1.01002200	-0.77494300
C	-0.12636600	3.70831600	-0.27551700
C	3.81650900	-0.54312700	-0.81665000
C	5.32681600	-0.41352900	-0.61223600
C	6.05391500	-0.85331700	-1.89453800
C	5.74309800	1.02278500	-0.27830700
C	5.73007300	-1.33148200	0.55682900
H	5.83463600	-1.89946000	-2.15539800
H	5.76656500	-0.22009200	-2.74872600
H	7.14304100	-0.76574700	-1.75686200
H	5.28525000	1.37714700	0.65813800
H	6.83508300	1.06761000	-0.14926800
H	5.47406000	1.72510100	-1.08261500
H	6.81249300	-1.25086700	0.74411200
H	5.20170900	-1.04907700	1.48146100
H	5.50387300	-2.38740500	0.34400000
C	0.24541800	5.15919600	0.03559000
C	1.21005900	5.67336300	-1.04766800
C	-0.98715400	6.06884900	0.07063100
C	0.93465700	5.20711600	1.41123000
H	2.14247900	5.08986100	-1.08043500
H	0.74414800	5.63007600	-2.04469800
H	1.47961100	6.72131000	-0.84265700
H	-1.70495100	5.75752500	0.84535500
H	-0.67635400	7.09917400	0.30097900
H	-1.51064500	6.08867000	-0.89774000
H	1.19906200	6.24628000	1.66244000
H	0.26836400	4.82449300	2.20047000
H	1.86126100	4.61361800	1.42892500
C	-0.81555600	-0.86020600	2.07481700
C	-1.57034900	0.24993000	2.41259500
C	-1.29613300	-1.73773100	1.05717200
C	-2.83135400	0.48683800	1.82824600
H	-1.20070500	0.94076100	3.17456500
C	-2.56742600	-1.51544600	0.47170400
H	-0.81811700	-2.71416500	0.93199100
C	-3.33587800	-0.40151400	0.90235100
H	-3.41237800	1.35838400	2.13350100
H	-3.05141800	-2.30836700	-0.10187200
C	-4.68962500	-0.19989800	0.29200100

F	-4.61050900	0.04756800	-1.03097300
F	-5.46716200	-1.28662100	0.41813100
F	-5.35824700	0.82431300	0.83136900
C	0.43290600	-1.17828600	2.85734200
C	1.64671400	-1.73390800	2.11008800
H	0.74257400	-0.26659100	3.39361600
C	2.45109200	-2.34854100	3.24749000
H	2.20965400	-0.96456100	1.56676000
H	1.33891700	-2.51150200	1.39537400
H	3.12456400	-1.60065100	3.70663900
H	3.06903900	-3.20018500	2.92007300
O	1.50407800	-2.78999100	4.20971100
C	0.22117900	-2.29090400	3.89702800
H	-0.41432600	-3.09355100	3.47387000
H	-0.26774400	-1.91999000	4.81234200
Br	-1.51892000	-2.71702000	-2.57792800

10’

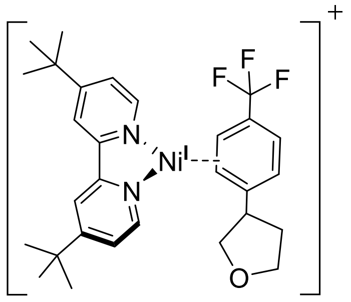

E= 3118.231649

G_{Corr}= 0.521495

C	0.16637900	2.35580700	-0.94552500
C	-1.33007100	0.72099500	-0.31509200
C	-0.85048000	3.28065300	-1.15039300
H	1.20970600	2.63194400	-1.11756100
C	-2.39770100	1.59218800	-0.49907400
H	-0.58082500	4.28161900	-1.48460900
H	-3.41289400	1.24601200	-0.30864500
C	-0.39669700	-2.64505200	0.67560500
C	-1.48896000	-0.68918300	0.13564400
C	-1.59166400	-3.29483600	0.96025600
H	0.55664600	-3.16936100	0.77809000
C	-2.72150800	-1.27483200	0.40172200
H	-1.55099000	-4.33361000	1.28477700
H	-3.63317700	-0.69139700	0.27778000
Ni	1.33464100	-0.32186100	-0.17504400
N	-0.06098800	1.10853800	-0.53721300
N	-0.34032700	-1.37698600	0.27217500
C	-2.80155800	-2.60882000	0.82458400
C	-2.17891000	2.90935500	-0.92535200
C	-3.36310400	3.85840200	-1.11812300
C	-4.32096900	3.25587800	-2.16143000
C	-4.09396900	4.01640800	0.22728900
C	-2.91362000	5.24105300	-1.60152100
H	-3.81377200	3.11248900	-3.12846900
H	-4.72422700	2.28419500	-1.83866800
H	-5.17371600	3.93408300	-2.32109000
H	-3.42283200	4.43153300	0.99558300
H	-4.94643100	4.70388500	0.11171700
H	-4.48592400	3.05689800	0.59733700
H	-3.79424400	5.88844100	-1.72968700
H	-2.24389100	5.73126900	-0.87811400
H	-2.39585100	5.18801300	-2.57172000
C	-4.16517800	-3.24110900	1.10897800
C	-5.00954900	-3.19586900	-0.17719400
C	-4.03513900	-4.69870300	1.56313700
C	-4.86589800	-2.43661300	2.21848200
H	-5.18335600	-2.16447000	-0.51942200
H	-4.51814600	-3.74931800	-0.99278800
H	-5.99270500	-3.65763200	0.00416800
H	-3.45200000	-4.78828900	2.49274400
H	-5.03610600	-5.11251700	1.75763700
H	-3.55993500	-5.32734200	0.79427700
H	-5.84510300	-2.88681900	2.44462000
H	-4.26912600	-2.43602000	3.14414400
H	-5.04055600	-1.39109100	1.92262100
C	3.09208700	0.03912100	1.12228900
C	2.98309000	-1.35550200	0.92655700
C	3.20630000	0.85813400	-0.02560600
C	2.94023800	-1.90772500	-0.36293100
H	2.88585100	-2.01378200	1.79156700
C	3.16437800	0.31537600	-1.31830400
H	3.28164400	1.94140800	0.08532900
C	3.01279200	-1.07283900	-1.49361700
H	2.80930000	-2.98435400	-0.48241500
H	3.21117600	0.97898400	-2.18240600
C	2.97222200	-1.67405400	-2.87168400
F	2.11706700	-2.70036300	-2.93677000
F	2.59843500	-0.78436800	-3.79461100
F	4.17117200	-2.14343700	-3.24078100
C	3.06282500	0.63033500	2.50472400
C	1.92266600	1.62534600	2.75384000
H	2.97011900	-0.19435000	3.22807300
C	2.42161800	2.34377300	3.99929800
H	0.95105200	1.13091000	2.89229800
H	1.83573100	2.32522700	1.90736200
H	2.11439200	1.80577900	4.91537600
H	2.04971800	3.37775800	4.07402300
O	3.83874700	2.35803200	3.90474800
C	4.27886000	1.48455300	2.88568700
H	4.64115200	2.06338700	2.01495100
H	5.11765900	0.87356000	3.25562900

11

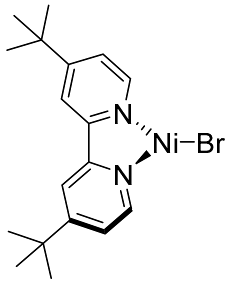

E=-4892.064635

G_{Corr}= 0.331608

C	0.95404600	2.61510300	0.03566700
C	-0.39563000	0.74530700	0.00395300
C	-0.14466300	3.46695400	0.02864500
H	1.97233200	3.01192100	0.05143800
C	-1.53890000	1.53515200	-0.00972300
H	0.03424600	4.54138700	0.04082400
H	-2.51997200	1.06007300	-0.03454700
C	0.92755800	-2.62517300	-0.03717500
C	-0.40318800	-0.74190200	-0.00382600
C	-0.17963600	-3.46588800	-0.02989000
H	1.94176700	-3.03227300	-0.05372500
C	-1.55443200	-1.52019600	0.01043700
H	-0.01166700	-4.54208100	-0.04278200
H	-2.53068800	-1.03529700	0.03625700
Ni	2.31829900	-0.01223400	-0.00032600
N	0.83937700	1.28674000	0.02378900
N	0.82631000	-1.29572300	-0.02465900

C	-1.46665300	-2.91994100	-0.00408500
C	-1.43711300	2.93393400	0.00404500
C	-2.70304000	3.79384400	-0.00935300
C	-3.50210300	3.48193800	-1.28730600
C	-3.55146500	3.45075400	1.22821200
C	-2.37829200	5.29112200	0.01590300
H	-2.90987400	3.70539400	-2.18863700
H	-3.80883400	2.42597200	-1.33263500
H	-4.41545700	4.09660500	-1.31840400
H	-2.99411200	3.64853900	2.15736200
H	-4.46397200	4.06730100	1.24036600
H	-3.86234800	2.39505200	1.23393400
H	-3.31447000	5.86973300	0.00651500
H	-1.82143500	5.57511400	0.92232400
H	-1.78933400	5.59837400	-0.86215600
C	-2.74086100	-3.76754100	0.00963400
C	-3.53631200	-3.44852900	1.28807400
C	-3.58648500	-3.41596700	-1.22748700
C	-2.43036900	-5.26783100	-0.01628300
H	-2.94562600	-3.67748500	2.18903700
H	-3.83346600	-2.38982200	1.33375200
H	-4.45518300	-4.05486900	1.31960900
H	-3.03126500	-3.61844600	-2.15690500
H	-4.50464400	-4.02407900	-1.23965300
H	-3.88764400	-2.35743900	-1.23259300
H	-3.37197000	-5.83755700	-0.00660300
H	-1.87669600	-5.55677900	-0.92309200
H	-1.84389700	-5.58095500	0.86137500
Br	4.61935000	-0.02085200	0.00050000

11’

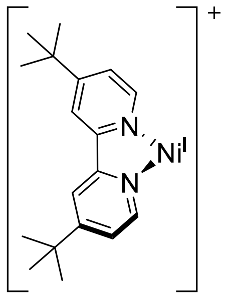

E=-2317.776951
G_{Corr}= 0.335532

C	2.64524300	1.85242300	-0.05868100
C	0.74564100	0.53894600	-0.00664300
C	3.47083600	0.73977000	-0.04501800
H	3.06200600	2.86178900	-0.08611400
C	1.51711200	-0.62175100	0.01568000
H	4.55129900	0.89008200	-0.06387900
H	1.02102200	-1.58892100	0.05314700
C	-2.64524800	1.85242300	0.05812800
C	-0.74564000	0.53894500	0.00635200
C	-3.47083400	0.73976600	0.04458500
H	-3.06201700	2.86179000	0.08537000
C	-1.51710700	-0.62175800	-0.01586200
H	-4.55129800	0.89007700	0.06338000
H	-1.02100700	-1.58892800	-0.05320200
Ni	-0.00002200	3.19428700	0.00027100
N	1.31136500	1.76022400	-0.03959800
N	-1.31136400	1.76022600	0.03914000
C	-2.91287500	-0.54594300	0.00409600
C	2.91288100	-0.54593200	-0.00429900
C	3.81350500	-1.78260200	0.01587800
C	3.00513900	-3.08367500	0.06250300
C	4.68051200	-1.78309900	-1.25591100
C	4.71799300	-1.71693000	1.25949500
H	2.38012300	-3.14728000	0.96674600
H	2.35314500	-3.19493300	-0.81772000
H	3.69370900	-3.94195200	0.07501200
H	5.32766100	-0.89477100	-1.31195600
H	5.33114100	-2.67159600	-1.26537500
H	4.05456500	-1.80929400	-2.16179300
H	5.37190600	-2.60239300	1.29400200
H	5.36318700	-0.82549400	1.25223400
H	4.11919100	-1.69928900	2.18374200
C	-3.81349400	-1.78262000	-0.01578700
C	-4.71847100	-1.71698800	-1.25904400
C	-3.00512400	-3.08367900	-0.06272200
C	-4.67999700	-1.78310300	1.25635000
H	-5.36371300	-0.82559000	-1.25153700
H	-4.12003600	-1.69932700	-2.18352800
H	-5.37235600	-2.60248000	-1.29328500
H	-2.35283800	-3.19496800	0.81728000
H	-3.69368400	-3.94196700	-0.07503200
H	-2.38040500	-3.14723200	-0.96717400
H	-5.33062600	-2.67159900	1.26608900
H	-4.05368600	-1.80928300	2.16198100
H	-5.32711200	-0.89476500	1.31263800

MECP

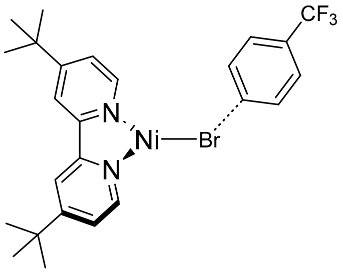

E(Singlet)= -5460.55925796
$G_{Corr-S=}$ 0.41677
E(Triplet)= -5460.5772203
$G_{Corr-T=}$ 0.409698

C	2.64524300	1.85242300	-0.05868100
C	0.74564100	0.53894600	-0.00664300
C	3.47083600	0.73977000	-0.04501800
H	3.06200600	2.86178900	-0.08611400
C	1.51711200	-0.62175100	0.01568000
H	4.55129900	0.89008200	-0.06387900
H	1.02102200	-1.58892100	0.05314700
C	-2.64524800	1.85242300	0.05812800
C	-0.74564000	0.53894500	0.00635200
C	-3.47083400	0.73976600	0.04458500
H	-3.06201700	2.86179000	0.08537000
C	-1.51710700	-0.62175800	-0.01586200
H	-4.55129800	0.89007700	0.06338000
H	-1.02100700	-1.58892800	-0.05320200
Ni	-0.00002200	3.19428700	0.00027100
N	1.31136500	1.76022400	-0.03959800
N	-1.31136400	1.76022600	0.03914000
C	-2.91287500	-0.54594300	0.00409600
C	2.91288100	-0.54593200	-0.00429900
C	3.81350500	-1.78260200	0.01587800
C	3.00513900	-3.08367500	0.06250300
C	4.68051200	-1.78309900	-1.25591100
C	4.71799300	-1.71693000	1.25949500
H	2.38012300	-3.14728000	0.96674600
H	2.35314500	-3.19493300	-0.81772000
H	3.69370900	-3.94195200	0.07501200
H	5.32766100	-0.89477100	-1.31195600

H	5.33114100	-2.67159600	-1.26537500
H	4.05456500	-1.80929400	-2.16179300
H	5.37190600	-2.60239300	1.29400200
H	5.36318700	-0.82549400	1.25223400
H	4.11919100	-1.69928900	2.18374200
C	-3.81349400	-1.78262000	-0.01578700
C	-4.71847100	-1.71698800	-1.25904400
C	-3.00512400	-3.08367900	-0.06272200
C	-4.67999700	-1.78310300	1.25635000
H	-5.36371300	-0.82559000	-1.25153700
H	-4.12003600	-1.69932700	-2.18352800
H	-5.37235600	-2.60248000	-1.29328500
H	-2.35283800	-3.19496800	0.81728000
H	-3.69368400	-3.94196700	-0.07503200
H	-2.38040500	-3.14723200	-0.96717400
H	-5.33062600	-2.67159900	1.26608900
H	-4.05368600	-1.80928300	2.16198100
H	-5.32711200	-0.89476500	1.31263800
